# A wheat ABC transporter contributes to both grain formation and mycotoxin tolerance

**DOI:** 10.1093/jxb/erv048

**Published:** 2015-03-01

**Authors:** Stephanie Walter, Amal Kahla, Chanemoughasoundharam Arunachalam, Alexandre Perochon, Mojibur R. Khan, Steven R. Scofield, Fiona M. Doohan

**Affiliations:** ^1^UCD Earth Institute and School of Biology & Environment Science, University College Dublin, Science Centre West, Belfield, Dublin 4, Ireland; ^2^USDA-ARS, Crop Production and Pest Control Research Unit and Purdue University, Department of Agronomy, 915 West Street, West Lafayette, IN 47907-2054, USA

**Keywords:** ABC transporter, breeding, deoxynivalenol, *Fusarium* head blight, grain formation, multidrug resistance, ripening, toxin resistance, wheat.

## Abstract

The mycotoxin deoxynivalenol (DON) acts as a disease virulence factor for *Fusarium* fungi, and tolerance of DON enhances wheat resistance to *Fusarium* head blight (FHB) disease. Two variants of an ATP-binding cassette (ABC) family C transporter gene were cloned from DON-treated wheat mRNA, namely *TaABCC3.1* and *TaABCC3.2*. These represent two of three putative genes identified on chromosomes 3A, 3B, and 3D of the wheat genome sequence. Variant *TaABCC3.1* represents the DON-responsive transcript previously associated with DON resistance in wheat. PCR-based mapping and *in silico* sequence analyses located *TaABCC3.1* to the short arm of wheat chromosome 3B (not within the FHB resistance quantitative trait locus *Fhb1*)*. In silico* analyses of microarray data indicated that *TaABCC3* genes are expressed in reproductive tissue and roots, and in response to the DON producer *Fusarium graminearum*. Gene expression studies showed that *TaABCC3.1* is activated as part of the early host response to DON and in response to the FHB defence hormone jasmonic acid. Virus-induced gene silencing (VIGS) confirmed that *TaABCC3* genes contributed to DON tolerance. VIGS was performed using two independent viral construct applications: one specifically targeted *TaABCC3.1* for silencing, while the other targeted this gene and the chromosome 3A homeologue. In both instances, VIGS resulted in more toxin-induced discoloration of spikelets, compared with the DON effects in non-silenced spikelets at 14 d after toxin treatment (≥2.2-fold increase, *P<*0.05). Silencing by both VIGS constructs enhanced head ripening, and especially so in DON-treated heads. VIGS of *TaABCC3* genes also reduced the grain number by more than 28% (*P<*0.05), both with and without DON treatment, and the effects were greater for the construct that targeted the two homeologues. Hence, DON-responsive *TaABCC3* genes warrant further study to determine their potential as disease resistance breeding targets and their function in grain formation and ripening.

## Highlight

Genes that enhance resistance to the Fusarium virulence factor deoxynivalenol (DON) are targets for disease resistance breeding. This study provides direct evidence that a wheat ABCC3 enhances resistance to DON.

## Introduction

ATP-binding cassette (ABC) transporters are transmembrane proteins that use the energy from ATP hydrolysis to transport substances across the cell membrane. ABC transporters are classified into distinct families according to their protein domain composition and organization (Verrier *et al.*, 2008). The ABC subfamily C (ABCC) transporters, also known as multidrug resistance-associated proteins (MRPs), were originally identified as vacuolar pumps of glutathione conjugates, but since then they have been implicated in a wide variety of functions, including detoxification, heavy-metal sequestration, chlorophyll catabolite transport, and ion-channel regulation (Wanke and Üner Kolukisaoglu, 2010).

*Fusarium* head blight (FHB) is a devastating disease of wheat that causes yield loss and the contamination of grain with the mycotoxin deoxynivalenol (DON) (Walter *et al.*, 2010). Resistance to initial infection (type I resistance) and resistance to the spread of the fungus within host tissue (type II resistance) (Schroeder and Christensen, 1963) are both widely accepted components of wheat resistance to FHB. Handa *et al.* (2008) identified a wheat *TaABCC* gene within a quantitative trait locus (QTL) on wheat chromosome 2D that enhances resistance to FHB disease. They highlighted this gene as a candidate for enhancing FHB resistance and reducing DON accumulation in grain. Walter *et al.* (2008) showed that the FHB resistance QTL on chromosome 3B of wheat (QTL *Fhb1*) regulates the transcription of another wheat *TaABCC* gene. The mycotoxin DON is a fungal virulence factor that facilitates disease spread *in planta*, causes premature bleaching of wheat spikelets, and thereby reduces grain yield (Lemmens *et al.*, 2005; Ansari *et al.*, 2007). Walter *et al.* (2008) showed that the regulation of *TaABCC* expression by DON was positively correlated with resistance to the toxin-induced bleaching of spikelets conferred by QTL *Fhb1.* QTL *Fhb1* is associated with resistance to disease spread and with the conversion of DON to the less toxic DON-3-glucoside (Lemmens *et al.*, 2005). Whether wheat ABCC proteins transport glycosylated DON into the vacuole remains to be determined, but the role of ABCC proteins in the wheat response to FHB and DON is likely to be more complex.

Here, we describe the cloning and mapping of the DON-responsive wheat ABCC transporter gene *TaABCC3.1* identified by Walter *et al.* (2008) and of its homologue *TaABCC3.2.* Using gene expression studies, we investigated whether *TaABCC3.1* is upregulated as an early response to DON or in response to the defence hormone jasmonic acid (JA). The temporal expression patterns of *TaABCC3.1* were investigated in order to test our previous hypothesis (Walter and Doohan, 2011) that differences in both the rapidity and magnitude of transcriptional responses are critical characteristics of genes involved in FHB resistance and DON tolerance. As part of virus-induced wheat gene-silencing experiments, we examined the effect of silencing *TaABCC3.1* or both *TaABCC3.1* and the chromosome 3A homeologue on toxin tolerance, ripening, and grain formation. Based on our findings, we discuss the potential of *TaABCC3* genes as targets of wheat breeding programmes.

## Materials and methods

### Plant material

Seeds of *Triticum aestivum* (wheat) cvs CM82036 and Remus were kindly supplied by Dr Hermann Buerstmayr (IFA-Tulln, Austria). Cultivar CM82036 is resistant to the discoloration of wheat heads induced by both FHB disease and DON treatment; FHB resistance and DON tolerance are conferred by the QTL *Fhb1* (on the short arm of chromosome 3B; synonym: *Qfhs.ndsu-3BS*), and *Qfhs.ifa-5A* (on chromosome 5A) also confers FHB resistance but not DON tolerance (Buerstmayr *et al.*, 2003). QTL *Fhb1* also confers resistance to DON-induced bleaching of wheat spikes of cv. CM82036 (Lemmens *et al.*, 2005). Cultivar Remus is susceptible to the discoloration of wheat heads caused by both FHB disease and DON treatment, and it carries neither QTL *Fhb1* nor QTL *Qfhs.ifa-5A* (Buerstmayr *et al.*, 2002).

### Adult wheat plant toxin experiments

Plants of wheat cvs CM82036 and Remus were grown under contained environment conditions, as described previously (Ansari *et al.*, 2007). At mid-anthesis (growth stage 64; Zadoks *et al.*, 1974), spikelets were treated with 15 μl of equimolar concentrations (i.e. 16.9mM) of either DON (5mg ml^–1^ in 0.2%, v/v, Tween 20; Sigma-Aldrich, USA) or cycloheximide (CHX) (4.7mg ml^–1^ in 0.2%, v/v, Tween 20); controls were treated with 0.2%, v/v, Tween 20. Treatments were injected into the three florets (15 µl treatment per floret) of four central spikelets per head. Treated spikelets were harvested at 4, 24, 48 or 72h post-treatment, flash frozen in liquid N_2_, freeze dried, and stored at –70 °C prior to RNA extraction. This experiment included three heads (one per plant) per treatment per time point per wheat cultivar and was conducted twice.

### Wheat seedling experiments

Seeds of wheat cv. CM82036 were germinated for 48h at 22 °C on moist Whatman No. 1 filter paper (Whatman, UK) and then placed in a new Petri dish on Whatman No. 1 filter paper soaked with 6ml of either 20 µg DON ml^–1^ in 0.2%, v/v, Tween 20 (i.e. 67.5 µM), 200 µM JA (Sigma-Aldrich) or 0.2%, v/v, Tween 20 (control) (12 seedlings per plate). Germinating seedlings were incubated at 20 °C under constant darkness for 1, 1.5, 2, 2.5, 3, 3.5, or 4h. The roots were then harvested, flash frozen in liquid N_2_, freeze dried, and stored at –70 °C prior to RNA extraction. Experiments included three replicates (plates) per treatment per time point. DON experiments were conducted twice and JA experiments three times.

### RNA extraction and cDNA synthesis

RNA was extracted as described by Ansari *et al.* (2007). DNase treatment of extracted total RNA was performed using a TURBO DNA-*free* ^TM^ kit (Ambion, USA), according to the manufacturer’s instructions. Reverse transcription of total RNA and real-time reverse transcription (RT)-PCR were performed as described by Walter *et al.* (2008).

### Gene cloning

A 587bp fragment of a DON-responsive wheat MRP ABC transporter from wheat cv. Frontana was identified previously within a wheat (cv. Frontana) cDNA library generated from DON-treated wheat roots (Walter *et al.*, 2008; GenBank accession no. FG985276). First-strand cDNA template was synthesised from 5 µg of total RNA using a Gene Racer^TM^ kit (Invitrogen, UK) and random primers (Invitrogen), according to manufacturer’s instructions. Both the *TaABCC3.1* and *TaABCC3.2* open reading frames (ORFs) were cloned via several steps of 5′ rapid amplification of cDNA ends (RACE) from cDNA generated from 4h DON-treated roots of wheat cv. CM82036 using the ABCC3 RACE primer sets indicated in Supplementary Table S1 at *JXB* online. For 5′ RACE, in some cases (see Supplementary Table S1) a nested PCR was performed subsequent to the first PCR in order to increase specificity. PCRs (25 µl volume) contained 0.5 µl of cDNA template (PCR round 1) or 0.5 µl of a 1:300 dilution of PCR round 1 PCR (nested PCR), 2 µM of each forward and reverse primer (Supplementary Table S1), 1.25U of TaKaRa LA Taq^TM^, and 1× LA buffer II (Mg^2+^ plus) (TaKaRa Bio, Japan), and 0.4mM of each dNTP. PCRs were performed in a Peltier thermal cycler DNA engine (MJ Research, UK) and the program for the first PCR consisted of: 2min of initial denaturation at 94 °C; five cycles of 94 °C for 30 s, and 72 °C for 4min; six cycles of 94 °C for 30 s and 70 °C for 4min; 30 cycles of 94 °C for 30 s and 68 °C for 4min; and a final extension step at 72 °C for 10min. The program for the nested PCR consisted of: 2min of initial denaturation at 94 °C, 30 cycles of 94 °C for 30 s and 68 °C for 3min; and a final extension step at 68 °C for 10min. The final *TaABCC3* ORF sequences were amplified with the ABCC3ORF primer sets indicated in Supplementary Table S1 using the same PCR conditions as for the first PCR of 5′ RACE. Amplified cDNA fragments were visualized as described by Walter *et al.* (2008) and cloned (pGEM-T Easy; Promega, UK); clones were transformed into *Escherichia coli* strain JM109 cells (Promega) and sequenced by Invitek GmbH (Germany) using T7 and SP6 primers. *TaABCC3.1* and *TaABCC3.2* mRNA and protein sequences are available in GenBank under accession numbers KM458975 and KM458976, respectively.

### Comparative, structural, and phylogenetic analyses

Generated sequence chromatograms were analysed with Geneious^®^ Pro 6.1.4 software (Biomatters, New Zealand). Vector sequences (identified via BLAST comparison with the Univec database at GenBank; Benson *et al.*, 2013) and primer sequences were trimmed. The ORF sequences of both *TaABCC3.1* and *TaABCC3.2* were assembled from all sequence chromatograms and downloaded transcript sequences in Geneious^®^ Pro 6.1.4 using the Geneious assembler with custom sensitivity settings of 2% maximum mismatches per read, up to 40% gaps per read, 400bp maximum gap size, word length 10, index word length 10, and maximum ambiguity 16.

Homologous wheat transcript and genome sequences were identified by a BLASTn search against *T. aestivum* transcriptome shotgun assemblies, expressed sequence tags and whole-genome shotgun contigs in GenBank (Benson *et al.*, 2013). All homologous sequences were imported into the Geneious^®^ Pro 6.1.4 software (Biomatters) and trimmed for vector sequences as described above before further analyses. For analyses of the exon–intron structure, *TaABCC3.1* and *TaABCC3.2* coding regions were mapped to the *T. aestivum* genome homologues using the Geneious assembler with custom sensitivity settings of 2% maximum mismatches per read, up to 60% gaps per read, 800bp maximum gap size, word length 10, index word length 10, and maximum ambiguity 16.

The three-dimensional structures of deduced *Ta*ABCC3 proteins were predicted using the protein structure prediction metaserver I-TASSER (Zhang, 2008; Roy et al., 2010, 2012). Protein parameters were calculated with PredictProtein (Rost *et al.*, 2004) and Expasy Compute pI/Mw (Sievers *et al.*, 2011).

Protein homologues of the proteins encoded by the *TaABCC3* ORFs were identified by BLASTP analysis against: (i) non-redundant proteins sequences in GenBank (Benson *et al.*, 2013); (ii) UniProtKB proteins identified with the HMMer web service (Finn *et al.*, 2011); and (iii) proteins in the WU-BLAST web server databases at EBI (Lopez *et al.*, 2003). ABCC-type (=MRP) ABC transporters were retrieved from the *Homo sapiens*, *Arabidopsis thaliana*, and all monocotyledonous plant protein databases at both GenBank (Benson *et al.*, 2013) and the UniprotKB database (The UniProt Consortium, 2012), using the respective species name and MRP ABC as search parameters. If available, GenBank protein identifiers were converted to Uniprot identifiers and the respective protein ID annotation was updated/corrected after searches in the UniProtKB database (The UniProt Consortium, 2012). TaABCC proteins were aligned with other ABCC proteins using the Clustal Omega tool (Goujon *et al.*, 2010; Sievers *et al.*, 2011).

Protein sequences were annotated for transmembrane helices and protein domains using InterProScan, the Transmembrane Prediction built into Geneious^®^ Pro 6.1.4, and TOPCONS (Bernsel *et al.*, 2009). Proteins that contained less than two transmembrane domains or nucleotide-binding fold, or proteins that lacked membrane helices upstream of the two transmembrane domains were considered as being either incomplete or belonging to a different subgroup of ABC transporters and were excluded from further analyses. Phylogenetic analysis was conducted using the two TaABCC proteins together and 185 full-length ABCC transporters. A dendrogram was constructed in MEGA4 (Tamura *et al.*, 2007), after calculation of the Dayhoff evolutionary distance matrix (Schwarz and Dayhoff, 1979), bootstrap values (1000 replicates), and an unrooted neighbour-joining tree (Saitou and Nei, 1987) from a multiple protein alignment generated with the Cobalt tool at NCBI (Papadopoulos and Agarwala, 2007). The accuracy of the Cobalt alignment was verified in Geneious^®^ Pro 6.1.4 by annotating the aligned proteins for transmembrane helices and protein domains as described above and inspecting the positioning of identified protein domains across the 187 full-sized proteins.

### Gene mapping studies

Mapping of the *TaABCC3.1* gene was performed as described previously by Walter *et al.* (2008) using the real-time RT-PCR primers for MRP (Walter *et al.*, 2008) (TaABCC3.1RT-F/R; Supplementary Table S1). *In silico* mapping was performed by a BLASTn search of *TaABCC3*.1 and *TaABCC3*.2 mRNA sequences against wheat chromosomes 3A, 3B, and 3D at the URGI database (http://wheat-urgi.versailles.inra.fr).

### Real-time RT-PCR analysis

Real-time RT-PCR analyses of *TaABCC3.1* and of housekeeping gene expression were performed in separate reactions as described by Walter *et al.* (2008) using the real-time RT-PCR primers for MRP and for the wheat housekeeping gene RNA helicase (clone ID: 1780) published previously by Walter *et al.* (2008) (Supplementary Table S1). For validation of virus-induced gene silencing (VIGS), the same RT-PCR conditions were used, but primers were designed such that they: (i) were not overlapping with the VIGS construct sequences; and (ii) specifically amplified the chromosome 3A, 3B (i.e. *TaABCC3.1*), or 3D variants (ABCC3VIGSRT-F/R; Supplementary Table S2 at *JXB* online). Specificity was confirmed via gene mapping using cv. Chinese Spring deletion lines, as described previously by Walter *et al.* (2008). All real-time RT-PCR analyses were conducted in duplicate. The fold changes in transcript accumulation relative to those of the respective housekeeping gene were calculated from the threshold cycle (*C*_T_) values obtained by real-time RT-PCR with the formula 2^– (*C*T target transcript – *C*T RNA housekeeping gene)^ (Livak and Schmittgen, 2001).

### VIGS

The barley stripe mosaic virus (BSMV)-derived VIGS vectors used in this study consisted of the wild-type BSMV ND18 α, β, and γ tripartite genome (Holzberg *et al.*, 2002; Scofield *et al.*, 2005). Two independent, non-overlapping gene fragments were used for VIGS of *TaABCC3* genes (see Supplementary Fig. S5 at *JXB* online) and these were amplified from pGEM-T::*TaABCC3.1* using primer pairs ABCC3VIGS1-F/R and ABCC3VIGS2-F/R (Supplementary Table S2). Once cloned into BSMV, they were respectively named BSMV:ABCCV1 and BSMV:ABCCV2. VIGS constructs BSMV:ABCCV1 and BSMV:ABCCV2 preferentially targeted *TaABCC3.1* for silencing; their homology and gene silencing specificity to the chromosome 3A, 3B (*TaABCC3.1*), and 3D homeologues is detailed in Table 1.

**Table 1.** Specificity of the constructs used for VIGS

Homeologue locus	Construct BSMV:ABCCV1		Construct BSMV:ABCCV2	
	Homology (%)^*a*^	Silencing^*b*^	Homology (%)^*a*^	Silencing^*b*^
3A	93	No	99	Yes
3B	100	Yes	100	Yes
3D	95	No	98	No

^*a*^Homology to the cv. Chinese Spring genome sequence.

^*b*^Based on allele-specific PCR analysis, using primers designed based on *TaABCC3.1* and wheat cv. Chinese spring genome sequence.

When cloning the ABCCV1 and ABCCV2 gene fragments, PCRs were performed with 30ng of plasmid DNA and 1 µM each of forward and reverse fragment-specific primers (Supplementary Table S2) in a 10 µl reaction containing 0.5U Taq DNA polymerase and 1× PCR buffer (Invitrogen), 1.5mM MgCl_2_, and 125 µM of each dNTP. PCRs were conducted in a Peltier thermal cycler DNA engine (MJ Research, USA) and the PCR program consisted of an initial denaturation step at 94 °C for 2min, 35 cycles of 94 °C for 30 s, and 60 °C for 30 s, and a final extension step at 72 °C for 5min. The amplified silencing fragments were cloned into the pGEM-T vector (pGEM-T Easy cloning kit; Promega). The pGEM-T vectors carrying the silencing fragments were digested with *Not*I and the inserts purified by gel extraction and then cloned into *Not*I-digested γ RNA vector pSL038-1 (Scofield *et al.*, 2005). The pSL038-1 plasmids harbouring the silencing fragments were sequenced by Macrogen (Korea) using the vector-specific primers pGamma-F/R (Supplementary Table S2). A BSMV γ RNA construct containing a 185bp fragment of the barley phytoene desaturase (*PDS*) gene served as a positive control for VIGS and has been described previously (Scofield *et al.*, 2005), with silencing resulting in premature bleaching of plants (results not shown).

The plasmids containing the BSMV β and γ genomes as well as γ RNA constructs with silencing fragments for *PDS* and *TaABCC3 w*ere linearized with *Mlu*I. The plasmid with BSMV α genome was linearized with *Spe*I. Capped *in vitro* transcripts were prepared from the linearized plasmids using an mMessage mMachine T7 *in vitro* transcription kit (Ambion, USA) following the manufacturer’s protocol. Flag leaves of plants of wheat cv. CM82036 at growth stage 47 (just before the emergence of the first wheat head; Zadoks *et al.*, 1974) were rub inoculated with BSMV constructs following the protocol described by Scofield *et al.* (2005). Rub inoculations were done with 1:1:1 mixtures of the *in vitro* transcripts of BSMV α, β, and γ RNA (BSMV:00) or derivatives of the γ RNA that contained barley *PDS* (BSMV:PDS4as), ABCC3V1 (BSMV: ABCC3V1), or ABCC3V2 (BSMV: ABCC3V2) fragments. At mid-anthesis (growth stage 65; Zadoks *et al.*, 1974), two central spikelets of heads of BSMV-infected plants were treated with 15 µl (30 µl per head) of either DON (5mg ml^–1^ in 0.2% Tween 20) or 0.2% Tween 20 (control) as described above. The third spikelet above the treated spikelet was sampled 24h after DON treatment, flash frozen in liquid N_2_, and stored at –70 °C prior to RNA extraction. Homeologue-specific RT-PCR assays were used to determine the impact of gene silencing on the transcription of chromosome 3A, 3B, and 3D gene variants (see above for RT-PCR analysis). The number of discoloured spikelets (including treated spikelets) was assessed at 14 d after DON inoculation and plants were visualized at 21 d after inoculation. At harvest (GD 90) heads were harvested and the mean grain weight and number of grain per head was determined. Sixteen heads (8 plants) were subjected to each treatment combination in each of two replica experiments.

### Data analysis

Datasets for the visual symptoms scored at 14 d after DON inoculation (number of discoloured spikelets), and grain number and some gene expression datasets were not normally distributed, as determined using the Johnson transformation test within Minitab^®^. Non-normally distributed data were analysed using the Kruskal–Wallis test within SPSS (SPSS Inc.). Normally distributed gene expression data were analysed using analysis of variance incorporating the Tukey’s pairwise comparison test at the 5% level of significance within Minitab^®^.

## Results

### Cloning and characterization of two wheat *TaABCC3* genes and their encoded proteins from wheat cv. CM82036

5′ RACE was used to clone the full-length ORF from DON-treated roots of the FHB/DON-resistant wheat cv. CM82036 (harvested at 4h after DON treatment), starting with the sequence of the DON-responsive wheat *TaABCC* transcript identified by Walter *et al.* (2008) (GenBank accession no. FG985276). Sequence assembly and subsequent phylogenetic analyses deduced that the cloned sequences represented two distinct homeologous wheat *ABCC* genes (GenBank accession nos KM458975 and KM458976), designated *Triticum aestivum ABC* transporter *C* family member *3* protein *1* (*TaABCC3.1*) and protein *2* (*TaABCC3.2*), respectively. The *TaABCC3.1* gene comprised 4503bp and shared 97% identity (e=0.0) with the 4494bp *TaABCC3.2* gene. The *TaABCC3.1* and *TaABCC3.2* genes encoded proteins of 1500 and 1497 aa, respectively (Supplementary Fig. S1 at *JXB* online) (See Supplementary Results at *JXB* online for further characterization of the genes, encoded protein structures, and models). Despite their high protein sequence identity, the predicted three-dimensional models of TaABCC3.1 and TaABCC3.2 were clearly distinct (Supplementary Fig. S2 at *JXB* online). The modelled hydrophobic N-terminal transmembrane domain 0 (TMD0) contained the majority of amino acid residues that putatively distinguish the TaABCC3.1 from the TaABCC3.2 protein (Supplementary Fig. S2G, H). The absence of a putative EAA motif (L-loop) in the TaABCC3 proteins defines them as putative ABC exporters rather than uptake systems (Saurin and Dassa, 1994; Biemans-Oldehinkel *et al.*, 2006).

**Fig. 1.**
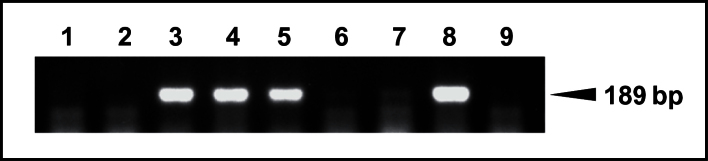
Localization of the DON-responsive *TaABCC3.1* gene to the short arm of wheat chromosome 3B. PCR analysis (performed using *TaABCC3.1* gene-specific PCR primers) of genomic DNA extracts of wild-type and 3BS deletion mutants of cv. Chinese Spring determined the 3BS chromosomal location of *TaABCC3.1*. Lanes: 1–5, Chinese Spring chromosome 3BS deletion mutants derivatives TA4524L1, TA4524L4, TA4524L2, TA4524L7, and TA4524L3, respectively (fragment length of retained chromosome 3BS=0.33, 0.55, 0.56, 0.75 and 0.87, respectively); 6, Chinese Spring 3BS ditelosomic line (Dt3BS) (accession no. GSTR36); 7, Chinese Spring 3B nullisomic-tetrasomic line (N3B-T3D) (accession no. GSTR73); 8, Chinese Spring (accession no. Cltr 14108); 9, water (negative control). Arrow: *TaABCC3.1* transcript fragment (expected product size: 189bp).

*TaABCC3.1* represents the original DON-responsive transcript (GenBank accession no. FG985276). FG985276 shared 99.49% identity with the *TaABCC3.1* mRNA but only 88.49% identity (e=0.0) with the *TaABCC3.2* mRNA. PCR primers specific to *TaABCC3.1* mapped to the wheat chromosome 3B bin 0.55–0.56, as determined by PCR-based mapping using chromosome deletion lines of cv. Chinese Spring (Fig. 1). *TaABCC3.1* also shared 100% identity (over 2055 nt, e=0.0) with the chromosome 3B-derived BAC clone TA3B63B13 (GenBank accession no. AM932681.1) (Charles *et al.*, 2008)*. In silico* analyses via a BLASTn search of the URGI database revealed that *TaABCC3.1* mRNA shared 98, 99, and 97% identity, respectively, with wheat cv. Chinese Spring contigs that map to wheat chromosomes 3A, 3B, or 3D. Hence, the homologous BAC clone, PCR mapping, and *in silico* mapping revealed 3B as the chromosomal location of the *TaABCC3.1* gene. The *TaABCC3.2* gene was 100% identical to another BAC clone (GenBank accession no. AC232259.1) (over 4491bp, e=0.0) whose chromosomal location has yet to be defined. *TaABCC3.2* the mRNA sequence from cv. CM82036 shared 97, 98, and 100% identity with cv. Chinese Spring contigs that map to chromosomes 3A, 3B, and 3D, respectively.

### Phylogenetic relationships between wheat *Ta*ABCC3 proteins and other ABC family C transporters

**Fig. 2.**
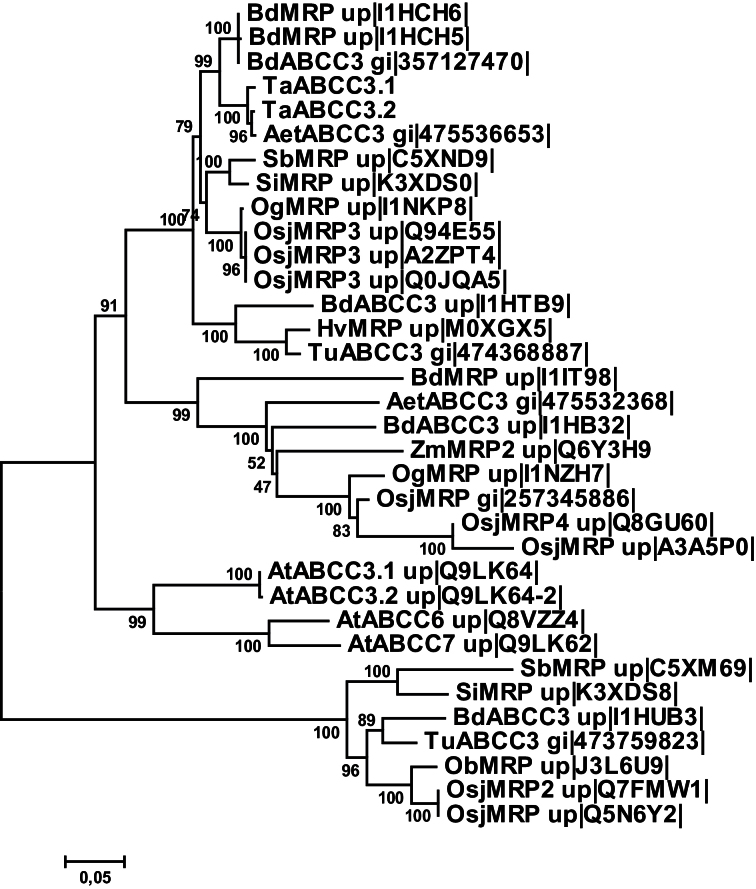
Evolutionary relationships of 34 ABCC-type ABC transporters, representing subclade IIA of plant clade II ABCC transporters. A phylogenetic dendrogram was generated in MEGA4 (Tamura *et al.*, 2007), after calculation of Dayhoff evolutionary distance matrix, bootstrap values (1000 replicates), and an unrooted neighbour-joining tree from a multiple protein alignment generated with the NCBI Cobalt tool (Papadopoulos and Agarwala, 2007). Bootstrap values are shown next to branches and depict the percentage of replicate trees in which the associated proteins clustered together in the bootstrap test (1000 replicates). For bootstrap values of <50, branches are collapsed. The tree is drawn to scale, i.e. branch lengths correlate directly with the Dayhoff evolutionary distances used to infer the phylogenetic tree. Species names were abbreviated as follows: Aet- *Aegilops tauschii* (Tausch’s goatgrass); At, *Arabidopsis thaliana* (mouse-ear cress); Bd, *Brachypodium distachyon* (purple false brome); Hv, *Hordeum vulgare* subsp. *vulgare* (barley); Ob, *Oryza brachyantha*; Og, *Oryza glaberrima* (African rice); Osj, *Oryza sativa* Japonica group (rice); Sb, *Sorghum bicolor* (sorghum); Si, *Setaria italica* (foxtail millet); Ta, *Triticum aestivum* (wheat); Tu, *Triticum urartu*; Zm, *Zea mays* (corn). Proteins were abbreviated either as ABCC (ABC transporter C family protein) or MRP (multidrug resistance-associated protein ABC transporter), with ABCC reflecting a more accurate annotation status. Except for the two newly described TaABCC3 proteins, all protein abbreviations are followed by their respective protein identifiers in the UniProtKB (up) (The UniProt Consortium, 2012) or GenBank (gi) (Benson *et al.*, 2013) database.

Phylogenetic analysis was conducted in order to: (i) infer the phylogenetic relationship between TaABCC3.1, TaABCC3.2, and other ABCC transporters; (ii) identify the closest protein homologues; and (iii) facilitate annotation of the two wheat transporters with regard to function and localization. Phylogenetic (MEGA4) analysis was conducted using TaABCC3.1, TaABCC3.2, and 185 full-sized ABCC proteins from 12 monocotyledonous plant species, *A. thaliana*, and humans. These proteins separated into at least two distinct clades, analogous to *A. thaliana* ABCC transporter clades I and II (Kolukisaoglu *et al.*, 2002) (Supplementary Fig. S3 at *JXB* online). TaABCC3.1 and TaABCC3.2 belonged to clade II, which also included *A. thaliana* clade II members (i.e. AtABCC3, -4, -5, -6, -7, -8, -9, -10 and 14) (Kolukisaoglu *et al.*, 2002) and ABCC/MRP proteins from cereal species, but none from humans. Based on the phylogenetic clustering and annotation, clade II was further subdivided into seven subclades, IIA–IIG (Supplementary Fig. S3). Both TaABCC3.1 and TaABCC3.2 belonged to the same ABCC subclade (IIA) as the vacuolar membrane-localized *A. thaliana* protein AtABCC3 (Szponarski *et al.*, 2003) (Fig. 2). ABCC3 proteins from *Aegilops tauschii* and *Brachypodium dystachion* were the closest homologues to both TaABCC3 proteins, as deduced by both phylogenetics and sequence homology (Fig. 2, sequence homology results not shown).

### *TaABCC3* genes are expressed in reproductive tissue and as an early response to treatment with fungus or fungal toxin

*In silico* analysis of published microarray data was conducted to investigate both the developmental and FHB disease response profile of *TaABCC3* transcripts. Based on the developmental study of Schreiber *et al.* (2009), the expression of *TaABCC3* transcripts peaked in wheat floral bracts, pistils, and developing embryos (Supplementary Fig. S4A at *JXB* online), suggesting that they play a role in reproductive tissue. Transcript levels also peaked in the roots and embryos of germinating wheat seeds. *In silico* analysis of the wheat microarray reported by Jia *et al.* (2009) showed that *TaABCC3* transcription was upregulated by *F. graminearum*, and that the presence of QTL *Fhb1* did not alter fungal activation of gene expression (Supplementary Fig. S4B). Boddu *et al.* (2007) conducted a microarray experiment that compared the effect of wild-type *F. graminearum* and a trichothecene-negative mutant derivative on the barley transcriptome. *In silico* analysis of that dataset highlighted that barley ABCC3 proteins were upregulated in wild type compared with in mutant-treated barley plants (Supplementary Fig. S4C).

**Fig. 3.**
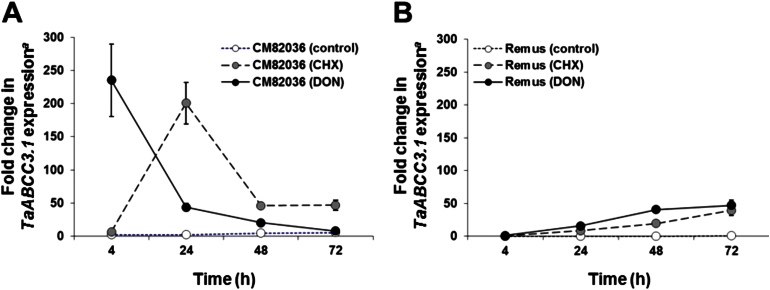
Temporal expression of the wheat *TaABCC3.1* transcript in spikelets of wheat cultivars CM82036 (A) and Remus (B) in response to DON and CHX treatment. Four central spikelets of the wheat cultivars CM82036 and Remus were treated with DON (5mg ml^–1^ in 0.2% Tween 20), CHX (4.7mg ml^–1^ in 0.2% Tween 20), or 0.2% Tween 20 at mid-anthesis. RNA extracted from treated spikelets at 4, 24, 48, or 72h post-treatment was used for real-time RT-PCR analysis (three replicates per cultivar per treatment from each of two independent experiments). ^*a*^Gene expression was quantified relative to the RNA helicase housekeeping gene [2^(–*C*T target)^/2^(–*C*T RNA helicase)^]. Results indicate means±standard error of the mean (SEM).

A *TaABCC3.1*-specific real-time RT-PCR assay was used to analyse the effect of DON (16.9mM) on the gene expression profile in spikelets of wheat genotypes that were either toxin resistant (cv. CM82036) or susceptible (cv. Remus) (Fig. 3). The effect of DON was compared with that of an equimolar amount of CHX; both compounds bind to the 60S ribosomal subunit, but CHX is a more potent inhibitor of protein synthesis (McLaughlin *et al.*, 1977; Masuda *et al.*, 2007). The basal accumulation of *TaABCC3.1* transcript in mock-treated samples was relatively constant over time, and higher in cv. CM82036 than in cv. Remus (6- to 21-fold; *P*≤0.05), but was very low compared with the levels detected in either DON- or CHX-treated samples (Fig. 3). The DON effect was more immediate in cv. CM82036 than in cv. Remus (Fig. 3). In cv. CM82036, *TaABCC3.1* accumulation in response to DON peaked at 4h post-treatment (101-fold induction by DON; *P*≤0.05), and was 5-fold higher than the maximal levels detected in DON-treated cv. Remus (at 72h post-treatment) (*P*≤0.05). In cv. Remus, the effect of DON on *TaABCC3.1* expression was more pronounced than the effect of CHX (Fig. 3). In cv. CM82036, the effect of DON was more immediate than that of CHX; at 4h post-treatment, the *TaABCC3.1* transcript levels in DON-treated samples were 34 times higher than in CHX-treated samples (*P*≤0.05).

**Fig. 4.**
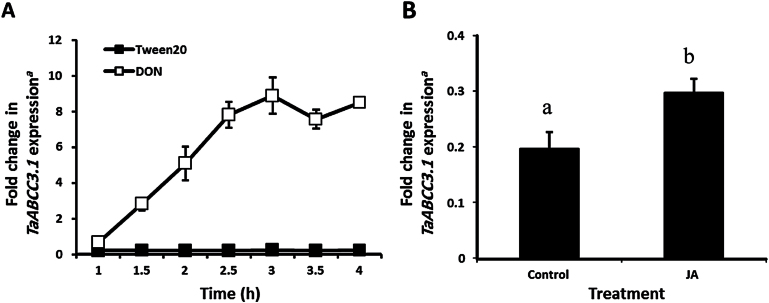
Expression of wheat *TaABCC3.1* transcript in roots of wheat cultivar CM82036 in response to DON (A) and JA (B) treatment. (A) Roots were treated with DON (20 μg ml^–1^ in 0.2% Tween 20) or 0.2% Tween 20 (controls) at 48h post-germination. RNA extracted from roots at 1, 1.5, 2, 2.5, 3, 3.5, and 4h post-treatment was used for real-time RT-PCR analysis (three replicates per treatment from each of two independent experiments). (B) Roots were treated with JA (200 µM) or 0.2% Tween 20 (controls) at 48h post-germination. RNA extracted from roots at 4h post-treatment was used for real-time RT-PCR analysis (three replicates per treatment from each of three independent experiments). ^*a*^Gene expression was quantified relative to the RNA helicase housekeeping gene [2^ (–*C*T target)^/2^ (–*C*T RNA helicase)^]. Results indicate means**±**SEM. Columns with a different letter are significantly different (*P*≤0.05).

Further evidence for a role of *TaABCC3.1* in the very early response to DON was obtained using wheat cv. CM82036 seedling roots exposed to a much lower concentration of the toxin (67.5 µM). Using the *TaABCC3.1-*specific RT-PCR assay, it was found that DON treatment significantly upregulated transcript accumulation in roots at all investigated time points (1–4h post-treatment) and peaked at 3h post-treatment (*P*≤0.05) (Fig. 4A). In further seedling studies, the FHB defence hormone JA (Gottwald *et al.*, 2012) was shown to induce the transcription of *TaABCC3.1* in roots of cv. CM82036. At 4h post-treatment, 200 µM JA significantly upregulated *TaABCC3.1* transcription in roots by 1.5-fold relative to mock treatment (*P*≤0.05) (Fig. 4B).

### Inhibition of *TaABCC3.1* expression increases toxin sensitivity

**Fig. 5.**
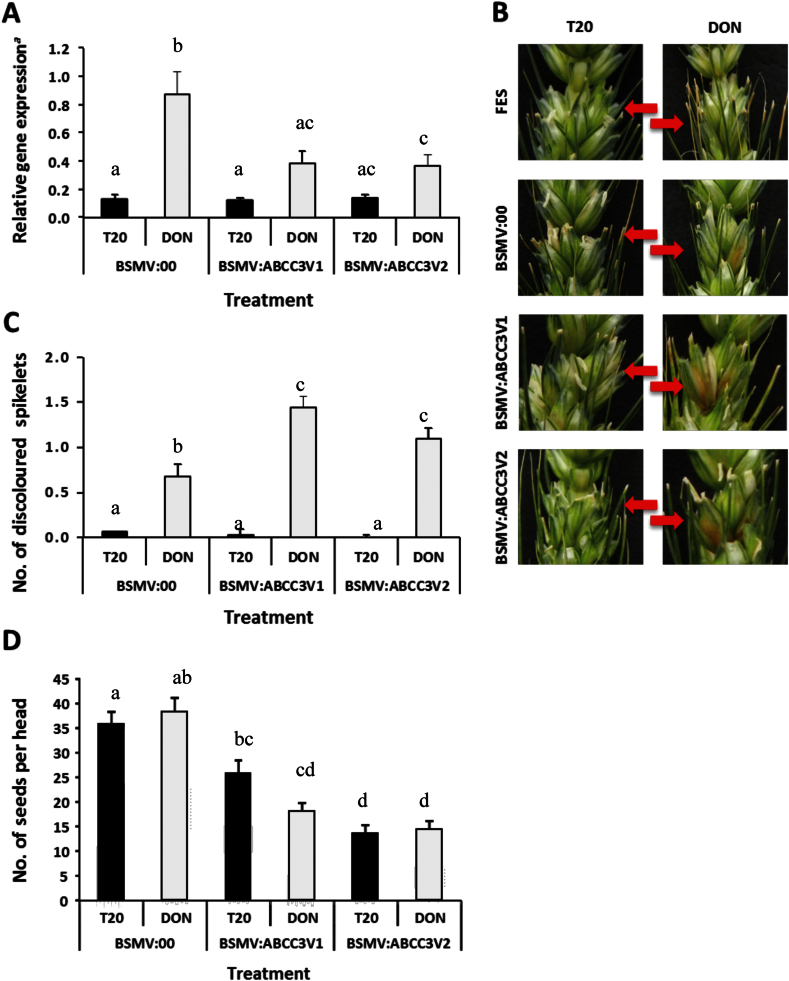
VIGS of *TaABCC3* genes in wheat. Flag leaves of DON-tolerant wheat cv. CM82036 were rub inoculated before the emergence of heads (growth stage 47; Zadoks *et al.*, 1974) with *in vitro-*transcribed RNAs representing either the empty vector BSMV:00, or one of the two constructs BSMV:TaABCC3V1 or BSMV:TaABCC3V2, which contain independent fragments of the *TaABCC3.1* gene. At mid-anthesis, the central spikelets were treated with either 5mg ml^–1^ of DON or Tween 20 (T20). (A) Relative gene expression. ^*a*^Gene silencing was quantified by real-time RT-PCR using primers specific to the *TaABCC3.1* variant on chromosome 3B (for the effect of VIGS on other variants please see Supplementary Fig. S6), relative to the RNA helicase housekeeping gene [2^(–*C*T target)^/2^(–*C*T RNA helicase)^]. (B) By 14 d after DON inoculation, DON-induced discoloration was more evident on gene-silenced compared with on mock (virus)-treated samples (red arrow indicates spikelets treated with either T20 or DON). (C) The degree of discoloration was quantified and the discoloration score for non-viral (mock)-treated samples was similar (*P>*0.10) to that for BSMV:00 (T20=0, DON=0.5). (D) To estimate yield, the number of grains per head was determined at the post-harvest stage. Results in (A), (C) and (D) indicate means±SEM calculated from 26–32 heads per treatment combination (10–16 heads per experiment). Columns with different letter are significantly different (*P*<0.05).

VIGS was performed to investigate the influence of TaABCC3.1 and homologous transporters on the phenotypic response of wheat cv. CM82036 to DON. The spikelets of this genotype are normally very resistant to DON-induced discoloration (Lemmens *et al.*, 2005; Ansari *et al.*, 2007). VIGS was performed using the two viral constructs BSMV:ABCC3V1 and BSMV:ABCC3V2, which, respectively, carry two non-overlapping *TaABCC3.1* gene fragments of 235 and 229bp (Supplementary Fig. S5). The empty vector BSMV:00 served as a negative control. In order to reduce transcript levels in emerging wheat heads, VIGS constructs were rub inoculated onto the flag leaves prior to head emergence. At the flowering stage, the central spikelets of heads that had emerged from the VIGS-treated tillers were injected with DON (5mg DON ml^–1^ in 0.2% Tween 20) or mock treated with 0.2% Tween 20. Spikelets above those treated were harvested after 24h and RNA extracts were subjected to *TaABCC3.1-*specific RT-PCR analysis to determine the success of the VIGS treatment. The resulting gene expression data for *TaABCC3.1* are presented in Fig. 5A. DON treatment induced the expression of *TaABCC3.1* by 6.7-fold (*P<*0.05). Gene silencing using either BSMV:ABCC3V1 or BSMV:ABCC3V2 reduced DON-induced transcription of *TaABCC3.1* by ≥56% compared with spikelets treated with BSMV:00 (*P<*0.05, Fig. 5A). Both constructs had similar effects on *TaABCC3.1* transcript levels in DON-treated tissue (*P*>0.10). In the absence of DON treatment, neither BSMV:ABCC3V1 nor BSMV:ABCC3V2 reduced the *TaABCC3.1* transcript levels relative to BSMV:00 (Fig. 5A). However, the basal transcript levels were relatively low.

Note that although the VIGS fragments were 100% homologous to *TaABCC3.1*, they also shared homology with chromosome 3A and 3D homeologues (Table 1). Consequently, chromosome 3A and 3D homeologue-specific RT-PCR assays were developed to determine the specificity of the VIGS treatment. The results are presented in Supplementary Fig. S6 at *JXB* online. DON induced the expression of the chromosome 3A and 3D homeologues by at least 2.4-fold (*P<*0.05). The basal and DON-induced expression of the chromosome 3D variant was very low relative to the 3A and *TaABCC3.1* (3B) homeologues. Construct BSMV:ABCCV1 did not significantly silence the transcription of either the 3A or the 3D homeologue (Supplementary Fig. S6). While construct BSMV:ABCCV2 did not silence the 3D homeologue, it did reduce the transcription of the 3A variant by 44%. Thus, the results of allele-specific RT-PCR assays confirmed that silencing via BSMV:ABCCV1 is specific to *TaABCC3.1* and, via BSMV:ABCCV2, to both *TaABCC3.1* and, to a slightly lesser extent, to the 3A homeologue.

Plants treated with either BSMV:ABCC3V1 or BSMV:ABCC3V2 were more sensitive to DON-induced discoloration of spikelets than those treated with BSMV:00 (Fig. 5B, C), thus confirming a direct role of TaABCC3.1 in DON tolerance. This was most pronounced at 14 d after DON treatment. As stated above, cv. CM82036 is very resistant to DON-induced bleaching of heads, and plants treated with BSMV:00 showed very little discoloration, i.e. less than one spikelet per head showed a brown discoloration, the mean being 0.5 (Fig. 5B, C). Those treated with BSMV:ABCC3V1 or BSMV:ABCC3V2 showed up to two spikelets per head discoloured due to the toxin, the mean being 1.4 and 1.1, respectively. Thus, DON caused >2.2-fold more spikelet discoloration in plants undergoing silencing of *TaABCC3* genes compared with plants treated with BSMV:00 (*P*<0.05) (Fig. 5B, C).

Later at 21 d post-inoculation, it was obvious that the majority of plants treated with either BSMV:ABCC3V1 or BSMV:ABCC3V2 were at a more advanced ripening stage than those exposed to the mock treatment (BSMV:00), and this was more obvious for plants treated with BSMV:ABCCV2 compared with those treated with BSMV:ABCCV1 (Supplementary Fig. S7 at *JXB* online). This ripening effect was generally more accelerated in DON-treated compared with Tween 20-treated samples and was distinct from the DON-induced browning observed at earlier time points. The other striking finding in the VIGS experiments was the implication that *TaABCC3* genes play a role in grain formation. Although VIGS of either *TaABCC3.1* or *TaABCC3.1* plus the 3A homeologue did not result in any appreciable reduction in transcript levels in Tween 20-treated plants, it did reduce grain number by >53% (*P<*0.001) compared with plants treated with BSMV:00 and Tween 20 (Fig. 5D). VIGS did not significantly affect the mean grain weight.

## Discussion

We report the cloning of two homeologous wheat full-length ABC subfamily C transporters of ABCC subclade IIA from DON toxin-treated wheat cDNAs of wheat cv. CM92036. The *TaABCC3.1* gene is located on wheat chromosome 3B and *TaABCC3.2* represents a closely related homologue. *In silico* sequence analyses and gene expression studies confirmed there are two DON-responsive homeologues of *TaABCC3.1* located on chromosomes 3A and 3D. Phylogenetic and predictive model analyses of these genes has increased our understanding of wheat ABC transporters, and modelling suggested that *TaABCC3.1* and its homologue *TaABCC3.2* encode ABC exporters that differ in the N-terminal transmembrane domain 0 (TMD0). Homeologue-specific gene expression analysis in the VIGS experiment deduced that *TaABCC3.1* was the most DON-responsive variant and that gene silencing reduced DON tolerance. Previously, Walter *et al.* (2008) demonstrated that the DON-induced upregulation of *TaABCC3.1* expression was linked to the FHB and DON resistance QTL *Fhb1*. The fact that QTL *Fhb1* did not enhance *TaABCC3* transcription in near-isogenic lines in response to *F. graminearum* (Jia *et al.*, 2009) may be due to collective detection of all *TaABCC3* gene homeologues by the microarray probe and hence a masking of allele-specific expression patterns. The early expression of *TaABCC3.1* under DON stress might be important for the establishment of resistance, because according to the observations by Zhuang *et al.* (2013), QTL *Fhb1-*mediated FHB resistance is established during the first 60h of infection. Because DON acts as a virulence factor for *Fusarium* pathogens and because *TaABCC3.1* gene expression is regulated by both QTL *Fhb1* and DON, TaABCC3.1 might contribute to resistance to FHB disease. A recent scientific breakthrough uncovered an ABC subfamily G transporter (Lr34) protein that confers resistance in wheat to a broad spectrum of fungal pathogens (Krattinger *et al.*, 2009) and highlighted the fact that unique haplotypes of this transporter are associated with disease resistance (Krattinger *et al.*, 2011). However, in line with other plant ABC transporters (Martinoia *et al.*, 2002; Wanke and Üner Kolukisaoglu, 2010), TaABCC3 transporters might be multifunctional, because they also positively affect grain formation in wheat heads and modulate ripening of the heads.

The fungal virulence factor DON exerts many toxic effects on plant cells, among which are oxidative stress and subsequent damage of cellular membranes (Walter *et al.*, 2010; Arunachalam and Doohan, 2013). The plant transforms toxic metabolites via a multiple-phase degradation pathway (Edwards *et al.*, 2011). After hydroxylation or carboxylation (phase I), xenobiotic compounds become conjugated (phase II) with sugars, amino acids, or peptides, e.g. glutathione (GSH). In phase III, conjugated metabolites are transported by ABC transporters across the vacuole membrane in an energy-dependent manner, which permits the final phase of detoxification that takes place in the vacuole. The human ABCC hMRP1 can transport the *Aspergillus* mycotoxin aflatoxin B1 (Loe *et al.*, 1997). Boddu *et al.* (2007) speculated that the barley protein encoded by a trichothecene-responsive *MRP* transcript may be involved in the transport of trichothecenes into the vacuole. Arabidopsis AtABCC3, the closest characterized homologue of TaABCC3 proteins (this study), is localized within the vacuolar membrane (tonoplast) (Szponarski *et al.*, 2003). Supposedly, TaABCC3 proteins also reside in the tonoplast. AtABCC3 can transport both GSH conjugates and chlorophyll catabolites (Tommasini *et al.*, 1998). Recently, DON–GSH conjugate and its derived processing products, DON*-S*-cysteinyl-glycine and DON-*S*-cysteine, were discovered in wheat, and the authors claimed that this provides evidence for GSH-mediated metabolism of the toxin *in planta* (Kluger *et al.*, 2013). Plant ABC transporters are well known to contribute to the uptake and degradation of pollutants, such as heavy metals and metalloids, from the soil (Cobbett and Meagher, 2008). However, although AtABC C3 promoter-driven expression can be induced in both roots and shoots by a variety of heavy metals and metalloids, direct evidence for metal/metalloid transport was not found; rather, it was demonstrated to transport GSH conjugates and chlorophyll catabolites (Tommasini *et al.*, 1998; Zientara *et al.*, 2009). Equally, despite the fact that in wheat the upregulation of *TaABCC3.1* transcription in both roots and heads was associated with the early DON tolerance response, to date there is no evidence for a direct role of this protein in DON transport and detoxification.

It seems more likely that the TaABCC3.1 protein transports physiological metabolites and that this contributes indirectly to DON resistance. The observation that DON resistance correlates with low levels of DON-induced bleaching of chlorophyllous tissue (Ansari *et al.*, 2007), the close relationship between TaABCC3 and the chlorophyll catabolite transporter AtABCC3 (Tommasini *et al.*, 1998), and the fact that *TaABCC3* gene silencing led to enhanced DON-induced bleaching in wheat heads indicate *TaABCC3.1* playing a role in chloroplast catabolite turnover. QTL *Fhb1* is linked with both the early induction of JA biosynthesis genes (Kugler *et al.*, 2013) and the DON-induced expression of *TaABCC3.1* (Walter *et al.*, 2008), and the hormone JA can upregulate *TaABCC3.1* transcription. One can speculate that the rapid synthesis of the wheat TaABCC3.1 transporter protein in response to DON, intense light, or other stress factors can be facilitated by early jasmonate-dependent activation of gene expression and TaABCC3.1-mediated disposal of chlorophyll catabolites into the vacuole. This in turn would reduce chloroplast stress, prevent cell damage by the photodynamic action of chlorophyll catabolites (Takamiya *et al.*, 2000), and thereby support cell viability and resistance to such stress factors. This fits well with the observed positive effect of TaABCC3 transporters on wheat grain formation and the influence on head maturation, as evidenced in the VIGS studies and the gene expression data from Schreiber *et al.* (2009).

Like TaABCC3 proteins, other cereal ABC transporters such as wheat Lr34 (Risk *et al.*, 2013), barley HvABCD1 and HvABCD2 (Mendiondo *et al.*, 2014), and rice OsABCG15 (Niu *et al.*, 2013; Qin *et al.*, 2013) can impact on grain development in cereal plants. In the case of TaABCC3 transporters, the influence on ripening was more pronounced after DON treatment, which could implicate a general role of TaABCC3 transporters in the maintenance of cell viability. The spread of *Fusarium* fungi within wheat plants is associated with a necrotrophic lifestyle that is facilitated by DON (Walter *et al.*, 2010). The maintenance of cell viability would counteract the virulence enhancing effects of DON and thus retard *Fusarium* colonization of cereal host plants, which in turn will reduce toxin accumulation in wheat tissues. ABCC proteins often display more than one function, and closely related proteins have been shown to be functionally diverse (Wanke and Üner Kolukisaoglu, 2010). It is known that sugars play a critical role in grain set in cereals, and thus it would be interesting to determine if TaABCC3 proteins impact on sucrose or invertase levels in grain or whether they impact on sugar transport in cereals.

The VIGS construct that targeted both *TaABCC3.1* and the chromosome 3A homeologue for silencing had a greater effect on grain formation and maturation compared with the one that specifically targeted *TaABCC3.1* for silencing. This suggests that both homeologues have additive effects on these traits. Indeed, there is a possible functional redundancy between TaABCC3 proteins, which would mean that the potential contribution of any variant, including *TaABCC3.1*, would be compensated. However, we did not see evidence of this in the VIGS experiments in that the non-targeted 3D variant was not upregulated in tissue within which the transcription of other variants was reduced.

Several wheat and barley genes recently have been shown to contribute to FHB resistance and/or DON tolerance (Poppenberger *et al.*, 2003; Schweiger *et al.*, 2010; Schweiger *et al.*, 2013). To the best of our knowledge, *TaABCC3.1* is the first wheat *ABCC* transporter gene validated to contribute to disease resistance in wheat. Its potential as a target for enhancing FHB resistance via a genetic modification approach can be validated via the analysis of transgenic lines overexpressing *TaABCC3.1.* Building on this work, subsequent analysis of polymorphisms that distinguish *TaABCC3* alleles of resistant from those of susceptible genotypes, as demonstrated for wheat *Lr34* (Krattinger *et al.*, 2009), will determine whether *TaABCC3.1* is a valid marker for DON tolerance at the DNA level. Functional markers that selectively distinguish disease resistance-associated allelic variants may provide a valuable tool for marker-assisted selection for DON tolerance in cereal breeding programmes.

## Supplementary data

Supplementary data are available at *JXB* online.

Supplementary Table S1. Primers used for cloning, genetic mapping, and real-time RT-PCR expression studies.

Supplementary Table S2. Primers used in VIGS analysis.

Supplementary Results. Characterization and phylogeny of the *TaABCC3* genes and their encoded proteins.

Supplementary Fig. S1. Alignment and domain organization of the TaABCC3.1 and TaABCC3.2 protein sequences.

Supplementary Fig. S2. Predictive three-dimensional models of TaABCC3.1 and TaABCC3.2 transport protein structure.

Supplementary Fig. S3. Evolutionary relationships of 187 ABCC-type ABC transporters.

Supplementary Fig. S4. *In silico* analysis of *TaABCC3* transcript levels.

Supplementary Fig. S5. Illustration of the position of the fragments within the mRNA encoding the wheat *TaABCC3.1* ABC transporter on wheat chromosomes 3BS targeted for gene silencing and RT-PCR for VIGS studies.

Supplementary Fig. S6. Analysis of the effect of VIGS of *TaABCC3* in wheat on the transcription of chromosome 3A, 3B and 3D gene variants in mock and DON-treated wheat heads.

Supplementary Fig. S7. Effect of VIGS of *TaABCC3* genes on ripening in response to either DON or Tween 20 treatment.

Supplementary Data

## Abbreviations:

ABC: ATP-binding cassette
ABCC: ABC subfamily C
BSMV: barley stripe mosaic virus
CHX: cycloheximide
*C*_T_: threshold cycle
DON: deoxynivalenol
FHB: *Fusarium* head blight
GSH: glutathione
JA: jasmonic acid
MRP: multidrug resistance-associated protein
ORF: open reading frame
QTL: quantitative trait locus
SEM: standard error of the mean
VIGS: virus-induced gene silencing.
