# Supplementary material for: A wheat ABC transporter contributes to both grain formation and mycotoxin tolerance
Source: J Exp Bot. 2015 Mar 1;66(9):2583–93. doi: 10.1093/jxb/erv048 (PMC4986867; doi:10.1093/jxb/erv048)
Supplement: Supplementary Data [file supp_66_9_2583__index.html]

A wheat ABC transporter contributes to both grain formation and mycotoxin tolerance — A wheat ABC transporter contributes to both grain formation and mycotoxin tolerance — Supplementary Data 

# A wheat ABC transporter contributes to both grain formation and mycotoxin tolerance

## Supplementary Data

Data files

**Files in this Data Supplement:**

- Supplementary Data - Supplementary Data
