# Supplementary material for: A wheat ABC transporter contributes to both grain formation and mycotoxin tolerance
Source: J Exp Bot. 2015 Mar 1;66(9):2583–93. doi: 10.1093/jxb/erv048 (PMC4986867; doi:10.1093/jxb/erv048)
Supplement: Supplementary Data [file supp_erv048_jexbot137596_file001.pdf]

**Supplemental Table S1: Primers used for cloning, genetic mapping, and real-time RT-PCR expression studies.**

| **Primers**1 | **Primer sequence (5′→3′)** | **Application** |
|---|---|---|
| ABCC3RACE-R1[2] | GCCAGACTCACTCAAGAAGCCTCAAC | 5' cDNA race 1 (PCR) |
| ABCC3RACE-R2[2] | TACCTGGATCGATGATACCAGTTGGTAGACTG | 5' cDNA race 2 (PCR) |
| ABCC3RACE-R3[2] | ATCCACTTCGCTTTGATCAGTTGAAGCTCTAT | 5' cDNA race 2 (nested PCR) |
| ABCC3RACE-R4[2] | GATTTACCACTGCCTGTTCTTCCAACAATACC | 5' cDNA race 3 (PCR) |
| ABCC3RACE-R5[2] | GAGTGGAATCGAAGAAAGACATAGGAGCTCTG | 5' cDNA race 4 (PCR) |
| ABCC3RACE-R6[2] | AGTAGCTGTGCTATCAACACGAATGGTACGAG | 5' cDNA race 4 (nested PCR) |
| ABCC3RACE-R8[2] | GAGATCGTGCATGTACCATGAGAAGAGG | 5' cDNA race 5 (PCR) |
| ABCC3RACE-F1/ABCC3RACE-R2 | CTTCCTCAGCGTCCTCACCTTCTCCT**/** TACCTGGATCGATGATACCAGTTGGTAGACTG | Subcloning |
| ABCC3RACE-F2/ABCC3RACE-R7 | CTCTTCTCATGGTACATGCACGATCTCTG/ CCTGGATAGGCTTAAAGAGATTGTTTCACTGC | Subcloning |
| ABCC3RACE-F2/ABCC3RACE-R7 | CTTCCTCAGCGTCCTCACCTTCTCCT**/** CTACGCGTGCGTCGCTCTGATCGTGTACT | Subcloning |
| ABCC3ORF-F/ ABCC3ORF-R | GCTCCACTCACCCAGCCTGC/ ACCGCAACGGCTGTTCTGTTCA | Amplification of ORF |
| TaABCC3.1RT-F/ TaABCC3.1RT-R[3] | GCCAACGAATAGATCCTCCA**/** ACCACCTTCAGCCTTTTCCT | Real time RT-PCR, Gene mapping |
| RNAhelicase-F/ RNAhelicase-R[3] | GCACAGGGAATCGTCAAAGT/ CACACATAGCTTCATTCGTTCA | Real time RT-PCR (housekeeping gene) |

[1] Primers containing 'ABCC3' in the name were used for both *TaABCC3.1* and *TaABCC3.2*, whereas primers containing 'ABCC3.1' where specific for gene variant *TaABCC3.1*.

[2] In combination with random primers from the Invitrogen 5'RACE kit (Invitrogen, UK).

**[3]** Walter *et al*.(2008).

**Supplemental Table S2: Primers used in VIGS analysis.**

| Primers | Primer sequence (5′→3′) | Application |
|---|---|---|
| ABCC3VIGS1-F/ ABCC3VIGS1-R | ACTCACTCAAGAAGCCTCAACGGC/ ACTCACTCAAGAAGCCTCAACGGC | Amplification of gene fragment used to generate VIGS construct ABCC3V1 |
| ABCC3VIGS2-F/ ABCC3VIGS2-R | CATACAGGCCCTTTTCCGTA/ GACCTCATCTCCCAGCTGAC | Amplification of gene fragment used to generate VIGS construct ABCC3V2 |
| pGAMMA-F/pGAMMA-R | TGATGATTCTTCTTCCGTTGC/ TGGTTTCCAATTCAGGCATCG | Sequencing of gene fragments within ABCC3V1 and ABCC3V2 |
| ABCC3-3AF/ ABCC3-3AR | GTAGTTCGTTAAGGATCATGTAGAAAG/ CGGGTATCACACAGTACATCAAA | Real time RT-PCR of the chromosome 3A variant of *TaABCC3* |
| ABCC3-3BF/ ABCC3-3BR | GGACCATACACGCCGAAA/ ACTGAACCACTTCCACTTCATTT | Real time RT-PCR of the chromosome 3B variant of *TaABCC3* (i.e. *TaABCC3.1*) |
| ABCC3-3DF/ ABCC3-3DR | GTGCTCTCGGATCGGATCT/ TTTCGGCGTGTATGGTCC | Real time RT-PCR of the chromosome 3D variant of *TaABCC3* |
| RNAhelicase-F/ RNAhelicase-R[1] | GCACAGGGAATCGTCAAAGT/ CACACATAGCTTCATTCGTTCA | Real time RT-PCR (housekeeping gene) |
| GAPDH-F/GAPDH-R[2] | TCACCACCGACTACATGACC/ ACAGCAACCTCCTTCTCACC | Real time RT-PCR (housekeeping gene) |

[1] Walter *et al*. (2008).
[2] (Soltanloo *et al.*, 2010).

**Supplemental Results: Characterisation and phylogeny of the *TaABCC3* genes and their encoded proteins.**

By mapping all cloned and downloaded *TaABCC3.1* transcript sequences to the *TaABCC3.1* gene, the mRNA length was determined to be 5025bp. The 5' untranslated regions (UTR) and 3' UTR comprised 22bp and 500bp, respectively. After mapping of all cloned and downloaded *TaABCC3.2* transcript sequences (N=91) to the *TaABCC3.2* gene the mRNA length was determined to be at least 4992bp. No transcripts were found that indicated the length of the 5' UTR of *TaABCC3.2* but the 3'UTR was found to be 487bp long. The original *TaABCC* transcript (Walter *et al.*, 2008) shared only 88.49% identity (e=0.0) with the *TaABCC3.2* mRNA. By computational mapping of the *TaABCC3.1* or *TaABCC3.2* mRNA sequences to wheat genomic homologues, both *TaABCC3* genes were shown to consist of 10 exons and 9 introns. Exon lengths ranged from 64bp (exon 8) to 2419bp (exon 1 in TaABCC3.1). Except for exon 1 (at 5' end of mRNA), exon lengths were equal between *TaABCC3.1* and *TaABCC3.2*. Intron lengths ranged from 84 to 212bp (data not shown).

Encoded proteins *Ta*ABCC3.1 and *Ta*ABCC3.2 share 97% identity, and respectively have theoretical isoelectric points (pI) of 6.02 and 5.82, and molecular weights (Mw) of 163841.56 and 163554.23 Da. The dominating amino acids (aa) are the non-polar (hydrophobic) aa leucine (*Ta*ABCC3.1: 12.5%; *Ta*ABCC3.2: 12.6%) and alanine (*Ta*ABCC3.1 and *Ta*ABCC3.2: 10.3%), which reflects their function as membrane proteins. At the sequence level, these proteins show highest similarity to ABCC3 proteins from cereal species (Suppl. Fig. S1). The closest homologue is *Aet*ABCC3 from the wheat progenitor *Aegilops tauschii* [GenBank ID: gi|475536653; *Ta*ABCC3.1: 1229/1251aa (98%) identity, 7/1251aa (0%) gaps; *Ta*ABCC3.2: 1240/1252aa (99%) identity, 7/1252aa (0%) gaps]. ABCC homologs of *Ta*ABCC3 proteins could be identified neither from the wheat progenitors *T. turgidum* and *Ae. speltoides.* However, a partial (lacking TMD0 domain) homolog from *T. urartu* was identified (GenBank ID: gi|474033437), but was more distantly related than *Aet*ABCC3 [*Ta*ABCC3.1: 1203/1247 aa (96%) identity, 29/1247aa (2%) gaps; *Ta*ABCC3.2: 1203/1248aa (96%) identity, 29/1248aa (2%) gaps].

The *Ta*ABCC3 proteins encode the two core functional units of ABC transporters. Each functional unit consists of a hydrophobic, integral transmembrane domain (TMD) and a cytosolic loop corresponding to the nucleotide-binding fold (NBF) (units are designated TMD1-NBF1 and TMD2-NBF2). Other features unique to the ABCC-subfamily of ABC transporters and present in *Ta*ABCC3 proteins include a hydrophobic N-terminal transmembrane domain (TMD0), a cytoplasmic linker between TMD0 and TMD1 and a hydrophilic C-terminal extension (Sánchez-Fernández *et al.*, 2001) (Supplemental Fig. S1). With TOPCONS (Bernsel *et al.*, 2009) both *Ta*ABCC3 proteins were predicted to contain 5 transmembrane (TM) spans in TMD0, 6 TM spans in TMD1 and 6 TM spans in TMD2 (Supplemental Fig. S1). The length of the cytoplasmic linker connecting TMD0 and TMD1 is not conserved; it consists of between 55 and 120 aa in plant ABCC3-like proteins (data not shown), of 55 aa in *Ta*ABCC3 proteins (Supplemental Fig. S1) and of 125 aa in human HsABCC1 (data not shown). Both of the NBFs in the *Ta*ABCC3 proteins (NBF1 and NBF2) contain a Walker A, Walker B and a C motif that are characteristic and name-giving for ABC transporters. Both also contain other conserved motifs typical for ABC transporter NBFs, i.e. the D-, H- and Q-loop (Supplemental Fig. S1) (Deeley *et al.*, 2006), respectively named after highly conserved asparagine, histidine and glutamine residues.

Predictive modelling using the i-Tasser server (Gunnaiah *et al.*, 2012; Roy *et al.*, 2010; Zhang, 2008) was employed to infer the 3D structure of the *Ta*ABCC3.1 and *Ta*ABCC3.2 proteins. Structures of P-glycoprotein (Aller *et al.*, 2009; Jin *et al.*, 2012), an ABCG-type ABC transporter, served as best models, based upon which structures of both *Ta*ABCC3 proteins were predicted. However, since P-glycoprotein does not have a TMD0 domain the predicted three-dimensional structure of the TMD0 domains in *Ta*ABCC3.1 and *Ta*ABCC3.2 is less reliable than for the other functional domains. The best 3D models for *Ta*ABCC3.1 and *Ta*ABCC3.2 (Supplemental Fig. S3) have confidence (C) -scores of -1.35 and -1.25, respectively, whereby C-scores typically range from -5 to 2, with higher C scores being better. The 3D structure models (Supplemental Fig. S3) clearly reflect that the *Ta*ABCC3 proteins represent transmembrane proteins, with the two $\alpha$-helical domains TMD1 and TMD2 forming the ABC transporter-characteristic functional unit essential for localisation within the

membrane, substrate binding and substrate transport (Linton and Higgins, 2007). In order to check if sequence differences between *Ta*ABCC3.1 and *Ta*ABCC3.2 might have a significant impact upon substrate specificity, residues diverging between both proteins were mapped along the polypeptide chains in the 3D models (Supplemental Figs. S3G and S3H). As expected from sequence homology analyses (Supplemental Figs. S1 and S2) the majority of diverging residues mapped to the TMD0 domains of the *Ta*ABCC3 proteins. No residues located on the interior side of intra-membrane substrate-binding TM helices were differing between both proteins (Supplemental Figs. S3G and S3H).

```
TaABCC3.1      1 MPTVASASSSPFAAAIAEPAALPVFLRPALLHGLGAGAHLLLALAVAARLLFAPAHRGKE   60
                 MPT AS SSSPFAAA+AEP ALPV L+PALLHGLGAGAHLLLALAVAARLLFAPA RGKE
TaABCC3.2      1 MPTAASPSSSPFAAAMAEP-ALPVLLQPALLHGLGAGAHLLLALAVAARLLFAPAPRGKE   59

TaABCC3.1     61 SAAAV-RGGGIRFRWGQFAVRATWALAASEVFLGVYSLVSWYLDNSGTGGGAGWGAPDAV  119
                 SAAA  RG GIR RWGQFAVRATWALAASEVFLG YSLVSWYLDNSG    AGWGAPDAV
TaABCC3.2     60 SAAAPGRGSGIRLRWGQFAVRATWALAASEVFLGAYSLVSWYLDNSGE---AGWGAPDAV  116

TaABCC3.1    120 ADQADTAARAVAWLLLAAYLQLQYRGRGEERFAAPLKLWWALFLLLSVLALAVHAATSLS  179
                 ADQAD AARAVAWLLLAAYL LQYRGRGEERFAAPLKLWWALFLLLSVLA+AVHAAT+LS
TaABCC3.2    117 ADQADAAARAVAWLLLAAYLHLQYRGRGEERFAAPLKLWWALFLLLSVLAVAVHAATTLS  176

TaABCC3.1    180 YGLPVPALPWARDAVEVLAGVALLVAGFSANTTGGSASEEPLLNGASESRGDDTVDASLF  239
                 YGLPVPALPWARDAVEVLA VALLVAGFSA TTGGSASEEPLLNGASESRGDDTVDASLF
TaABCC3.2    177 YGLPVPALPWARDAVEVLAAVALLVAGFSAKTTGGSASEEPLLNGASESRGDDTVDASLF  236

TaABCC3.1    240 TSAGFLSVLTFSWMGPLLAVGNKKALGLDDVPDLDHADSVAGLLPSFKTNLEAQAGDGSG  299
                 TSAGFLSVLTFSWMGPLLAVGNKKALGLDDVPDLDHADSVAGLLPSFKTNLEAQAGDGSG
TaABCC3.2    237 TSAGFLSVLTFSWMGPLLAVGNKKALGLDDVPDLDHADSVAGLLPSFKTNLEAQAGDGSG  296

TaABCC3.1    300 PKFTAFKLTKALVRTVWWHIAVTALYALIYNLATYVGPYLIDSLVQYLNGDERYASKGKL  359
                 PKFTAFKLTKALVRTVWWHIAVTALYALIYNLATYVGPYLIDSLVQYLNGDE YASKGKL
TaABCC3.2    297 PKFTAFKLTKALVRTVWWHIAVTALYALIYNLATYVGPYLIDSLVQYLNGDEGYASKGKL  356

TaABCC3.1    360 LVVTFIVAKVFECLSQRHWFFRLQQAGIRARSALVSVVYQKGLSLSSISRQSRTSGEMIN  419
                 LVVTFIVAKVFECLSQRHWFFRLQQAGIRARSALVSVVYQKGLSLSS SRQSRTSGEMIN
TaABCC3.2    357 LVVTFIVAKVFECLSQRHWFFRLQQAGIRARSALVSVVYQKGLSLSSTSRQSRTSGEMIN  416

TaABCC3.1    420 IISVDADRVGLFSWYMHDLWLVPLQVGMALFILYSTLGVASLAALGATIVVMLANVPPMK  479
                 IISVDADRVGLFSWYMHDLWLVPLQVGMALFILYSTLGVASLAALGATIVVMLANVPPMK
TaABCC3.2    417 IISVDADRVGLFSWYMHDLWLVPLQVGMALFILYSTLGVASLAALGATIVVMLANVPPMK  476

TaABCC3.1    480 MQEKFQQKLMDCKDVRMKATSEILRNMRILKLQGWEMKFLSKIIDLRTTETSWLKKYLYT  539
                 MQEKFQQKLMDCKDVRMKATSEILRNMRILKLQGWEMKFLSKIIDLRTTETSWLKKYLYT
TaABCC3.2    477 MQEKFQQKLMDCKDVRMKATSEILRNMRILKLQGWEMKFLSKIIDLRTTETSWLKKYLYT  536
```

TMD0

CL

TMD1

```
                        ...................................
TaAbCC3.1    540 STAATFVFWGAPTFVAVVTFGACMLLGIPLESGKVLSALATFRVLQEPIYNLPDTISMMI  599   TMD1
                 STAATFVFWGAPTFVAVVTFGACMLLGIPLESGKVLSALATFRVLQEPIYNLPDTISMMI
TaAbCC3.2    537 STAATFVFWGAPTFVAVVTFGACMLLGIPLESGKVLSALATFRVLQEPIYNLPDTISMMI  596

TaAbCC3.1    600 QTKVSLDRIASFLCLEELPTDAVERLPSGSSNVAIEVSNGCFSWDGSPELPTLKDLNFEA  659
                 QTKVSLDRIASFLCLEELPTDAVERLPSGSSNVAIEVSNGCFSWDGSPELPTLKDLNF+A
TaAbCC3.2    597 QTKVSLDRIASFLCLEELPTDAVERLPSGSSNVAIEVSNGCFSWDGSPELPTLKDLNFQA  656

                         Walker A                          Q loop
TaAbCC3.1    660 QQGMRVAVCGTVGSGKSSLLSCILGEVPKLSGEVKTCGTMAYVSQTAWIQSGKIQDNILF  719
                 QQGMRVAVCGTVGSGKSSLLSCILGEVPKLSGEVK CGT AYVSQTAWIQSGKIQDNILF
TaAbCC3.2    657 QQGMRVAVCGTVGSGKSSLLSCILGEVPKLSGEVKICGTTAYVSQTAWIQSGKIQDNILF  716

                                                       C motif       Walker B
TaAbCC3.1    720 GKEMDSEKYDRVLEWCSLKKDLEILPFGDKTVIGERGINLSGGQKQRIQIARALYQDADI  779   NBF1
                 GKEMDSEKYDRVLEWCSLKKDLEILPFGDKTVIGERGINLSGGQKQRIQIARALYQDADI
TaAbCC3.2    717 GKEMDSEKYDRVLEWCSLKKDLEILPFGDKTVIGERGINLSGGQKQRIQIARALYQDADI  776

           Walker B  D loop
TaAbCC3.1    780 YLFDDPFSAVDAHTGSHLFKECLLGALASKTVVYVTHHIEFLPSADLILVMKGGRIAQAG  839
                 YLFDDPFSAVDAHTGSHLFKECLLGALASKTVVYVTH IEFLPSADLILVMKGGRIAQAG
TaAbCC3.2    777 YLFDDPFSAVDAHTGSHLFKECLLGALASKTVVYVTHQIEFLPSADLILVMKGGRIAQAG  836

TaAbCC3.1    840 KYNDILGSGEELMELVGAHQDALTALDVIDVANGGSETISSSLSRSLSSAEEKDKQSGKD  899
                 KYNDILGSGEELMELVGAHQDALTALDVIDVANGGSETIS SLSRSLSSAEEKDKQ+GKD
TaAbCC3.2    837 KYNDILGSGEELMELVGAHQDALTALDVIDVANGGSETISLSLSRSLSSAEEKDKQNGKD  896

                                           ......................................
TaAbCC3.1    900 NGDKVQSGQLVQEEEREKGRVGFWVYWKYLTLAYGGALVPFVLIAQLLFQVLQIASNYWM  959
                 +GDKVQSGQLVQEEEREKGRVGFWVYWKYLTLAYGGALVPFVLIAQLLFQVLQIASNYWM
TaAbCC3.2    897 DGDKVQSGQLVQEEEREKGRVGFWVYWKYLTLAYGGALVPFVLIAQLLFQVLQIASNYWM  956

                 ............................................................
TaAbCC3.1    960 AWASPVSKDAEPPVSTSTLIYVFVALAVASSLCILIRALFLVTAAYKTATLLFNKMHMAI 1019
                 AWASPVSKDAEPPVSTSTLIYVFVALAVASSLCILIRALFLVTAAYKTATLLFNKMHMAI
TaAbCC3.2    957 AWASPVSKDAEPPVSTSTLIYVFVALAVASSLCILIRALFLVTAAYKTATLLFNKMHMAI 1016   TMD2

                 ............................................................
TaAbCC3.1   1020 FRAPMSFFDSTPSGRILNRASTDQSEVDTNIAYQMGSVAFSIIQLMGIIAVMSQVAWQVF 1079
                 FRAPMSFFDSTPSGRILNRASTDQSEVDTNIAYQMGSVAFSIIQLMGIIAVMSQVAWQVF
TaAbCC3.2   1017 FRAPMSFFDSTPSGRILNRASTDQSEVDTNIAYQMGSVAFSIIQLMGIIAVMSQVAWQVF 1076
```

```
TaABCC3.1   1080 LVFVPVIIICFWYQRYYIETARELQRLVGVCKAPIIQHFAESITGSTTIRSFGKEHQFVS 1139
                 LVFVPVIIICFWYQRYYIETARELQRLVGVCKAPIIQHFAESITGSTTIRSFGKEHQFVS
TaABCC3.2   1077 LVFVPVIIICFWYQRYYIETARELQRLVGVCKAPIIQHFAESITGSTTIRSFGKEHQFVS 1136
                                                                                  TMD2

TaABCC3.1   1140 TNSHLMDAYSRPKFYNAAAMEWLCFRLDTLSSFTFAFALVFLISLPTGIIDPGIAGLAVT 1199
                 TNSHLMDAYSRPKFYNA AMEWLCFRLDTLSSFTFAFALVFLISLPTGIIDPGIAGLAVT
TaABCC3.2   1137 TNSHLMDAYSRPKFYNAVAMEWLCFRLDTLSSFTFAFALVFLISLPTGIIDPGIAGLAVT 1196


TaABCC3.1   1200 YGLNLNMLQAWVVWSMCNLENKIISVERILQYISIPEEPPLSMSEDKLPHNWPSQGEIQL 1259
                 YGLNLNMLQAWVVWSMCNLENKIISVERILQYISIPEEPPLS SEDKLP NWPS+GEIQL
TaABCC3.2   1197 YGLNLNMLQAWVVWSMCNLENKIISVERILQYISIPEEPPLSTSEDKLPSNWPSEGEIQL 1256

                                              Walker A
TaABCC3.1   1260 RDVHVRYAPQLPFVLKGLNVTFPGGMKTGIVGRTGSGKSTLIQALFRIVEPTVGQILVDG 1319
                 RDVHVRYAPQLPFVLKGLNVTFPGGMKTGIVGRTGSGKSTLIQALFRIVEPTVGQILVDG
TaABCC3.2   1257 RDVHVRYAPQLPFVLKGLNVTFPGGMKTGIVGRTGSGKSTLIQALFRIVEPTVGQILVDG 1316
                                                                                  NBF2
                                   Q loop
TaABCC3.1   1320 VDICTIGLHDLRSRLSIIPQDPTMFEGTVRSNLDPLNEYNDDQIWEALDNCQLGDEVRKK 1379
                 VDICTIGLHDLRSRLSIIPQDPTMFEGTVRSNLDPLNEYND+QIWEALDNCQL DEVRKK
TaABCC3.2   1317 VDICTIGLHDLRSRLSIIPQDPTMFEGTVRSNLDPLNEYNDNQIWEALDNCQLEDEVRKK 1376

                              C motif       Walker B  D loop
TaABCC3.1   1380 ELKLDSPVIENGENWSVGQRQLVCLGRVILKRTKILVLDEATASVDTATDNMIQKTLREN 1439
                 ELKLDSPVIENGENWSVGQRQLVCLGRVILKRTKILVLDEATASVDTATDNMIQKTLREN
TaABCC3.2   1377 ELKLDSPVIENGENWSVGQRQLVCLGRVILKRTKILVLDEATASVDTATDNMIQKTLREN 1436

                      H loop
TaABCC3.1   1440 FSEATVITIAHRITSVLDSDMVLLLDNGVAVERDTPAKLLENKSSLFSKLVAEYTIRATH 1499
                 FSEATVITIAHRITSVLDSDMVLLLDNGVAVERDTPAKLLENKSSLFSKLVAEYT+RATH
TaABCC3.2   1437 FSEATVITIAHRITSVLDSDMVLLLDNGVAVERDTPAKLLENKSSLFSKLVAEYTMRATH 1496

TaABCC3.1   1500 A* 1501
                  *
TaABCC3.2   1497 T* 1498
```

**Supplemental Figure S1: Alignment and domain organisation of the *Ta*ABCC3.1 and *Ta*ABCC3.2 protein sequences.** The two proteins share 97% identity (1463/1502 aa, gaps: 5/1502 aa, 0%). The motifs characteristic for ABC proteins (i.e. Walker A, Walker B and C motif) as well as other conserved motifs within the nucleotide binding folds (i.e. D-, H-, and Q-loop) (Deeley *et al.*, 2006) are coloured in grey. Putative transmembrane domains (TMD) are indicated with a dotted line and were predicted with Geneious® Pro 6.1.4 (Biomatters Ltd.,

New Zealand). Putative transmembrane spans within the TMDs are surrounded by grey boxes. The approximate position of all domains is summarised on the right hand side. Abbreviations: CL– cytoplasmic loop; TMD– transmembrane domain; NBF– nucleotide binding fold.

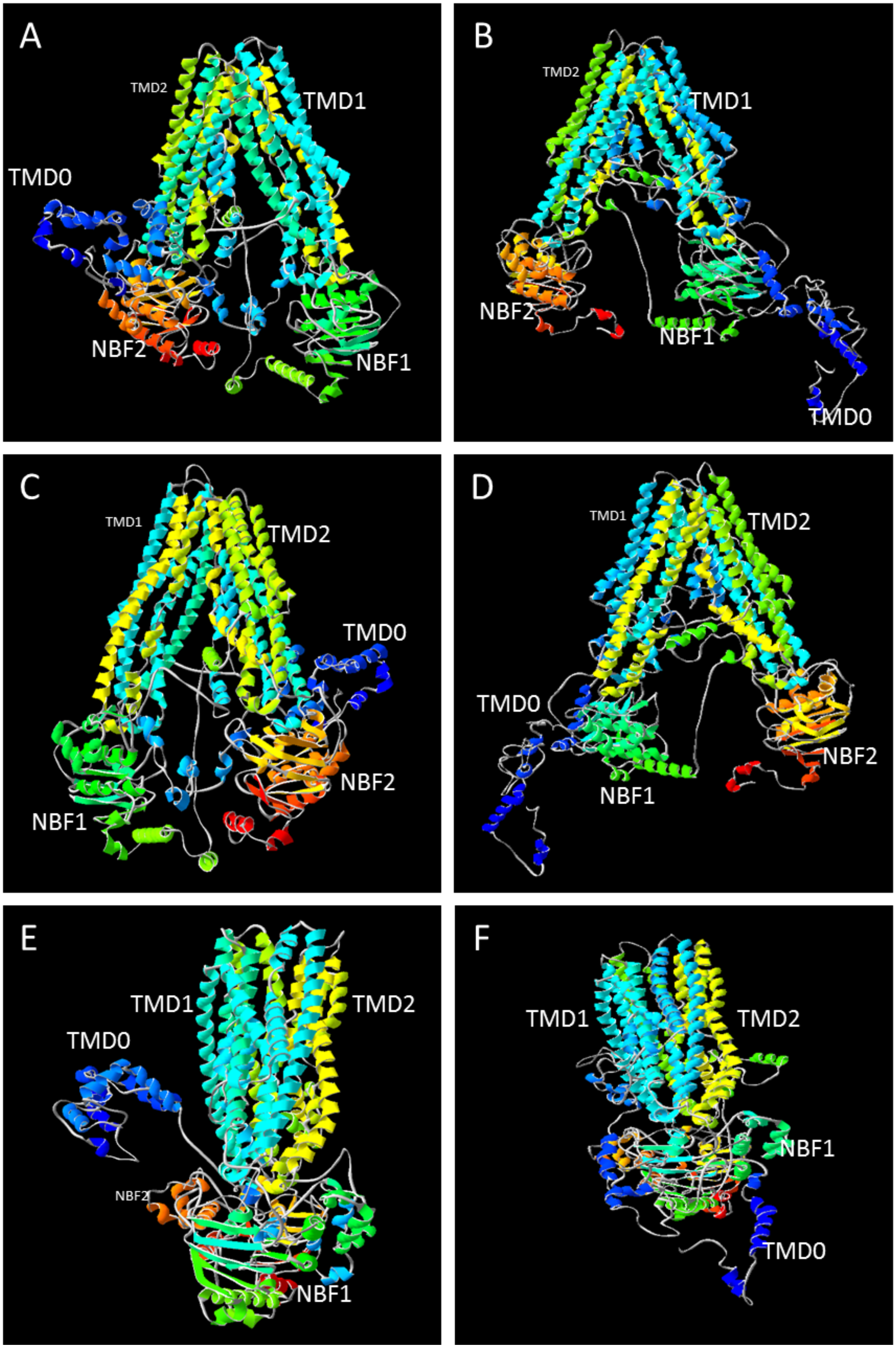
A
TMD2
TMD1
TMD0
NBF2
NBF1
B
TMD2
TMD1
NBF2
NBF1
TMD0
C
TMD1
TMD2
TMD0
NBF2
NBF1
D
TMD1
TMD2
TMD0
NBF2
NBF1
E
TMD1
TMD2
TMD0
NBF2
NBF1
F
TMD1
TMD2
NBF1
TMD0

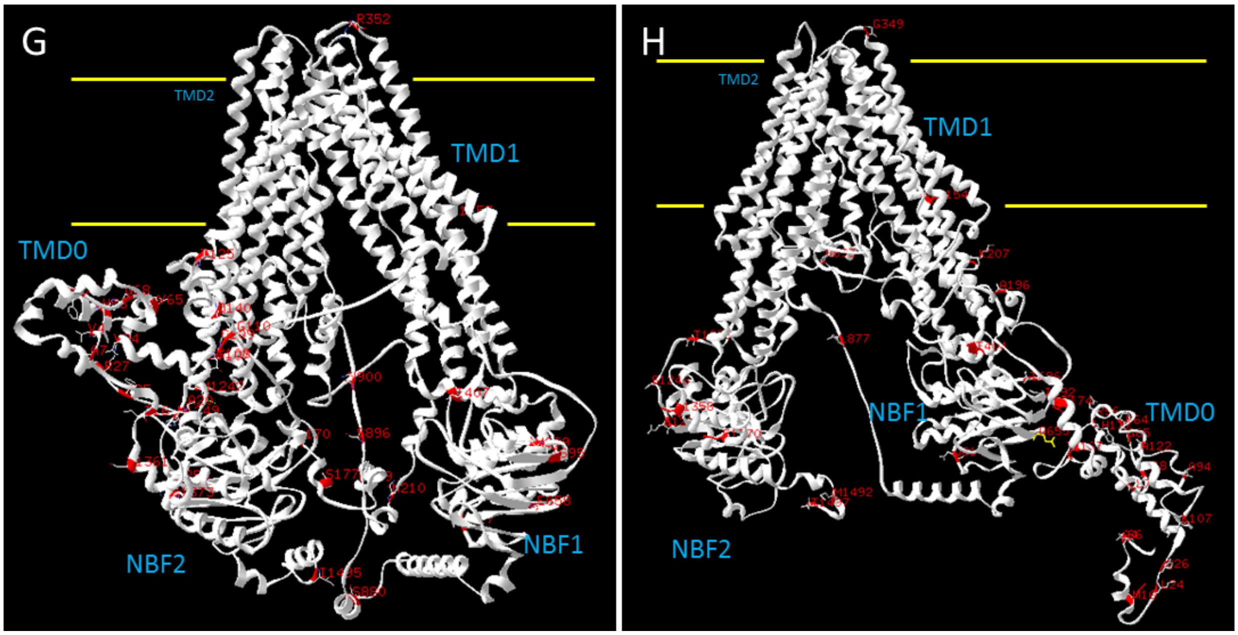


**Supplemental Figure S2: Predictive three-dimensional models of *Ta*ABCC3.1 and *Ta*ABCC3.2 transport protein structure. (A)** to **(H)**: Ribbon presentation of the 3D structures of *Ta*ABCC3.1 or *Ta*ABCC3.2 in open dimer conformation as predicted with the i-Tasser server (Roy *et al.*, 2010; Roy *et al.*, 2012; Zhang, 2008) based upon crystal and X-ray structures of P-glycoprotein (Aller *et al.*, 2009; Jin *et al.*, 2012). Approximate positions of functional protein domains are indicated in white (Figure A to F) or blue (Figure G and H), whereby domains located on the backside of the respective view are indicated with text. **(A)** to **(F)**: Amino acid residues are colour coded as a gradient along the polypeptide chain from N-terminus (blue) to the C-terminus (red). Each secondary structure element has a single colour, and random-coils are grey. **(A)** and **(B)**: Front view of *Ta*ABCC3.1 and *Ta*ABCC3.2, respectively. **(C)** and **(D)**: Back view of *Ta*ABCC3.1 and *Ta*ABCC3.2, respectively. **(E)** and **(F)**: Side view as seen from the NBF1 side of *Ta*ABCC3.1 and *Ta*ABCC3.2, respectively, to demonstrate structure of the TMD0 domains. **(G)** Front view of *Ta*ABCC3.1 protein resembling the front view in (A), whereby amino acid residues that differ from the homologous *Ta*ABCC3.2 protein are highlighted in red. Putative position of membrane layers are indicated with yellow lines. **(H)**: Front view of *Ta*ABCC3.2 protein resembling the front view in (B), whereby residues that differ from *Ta*ABCC3.1 are highlighted in red. Abbreviations: TMD– transmembrane domain; NBF– nucleotide binding fold.

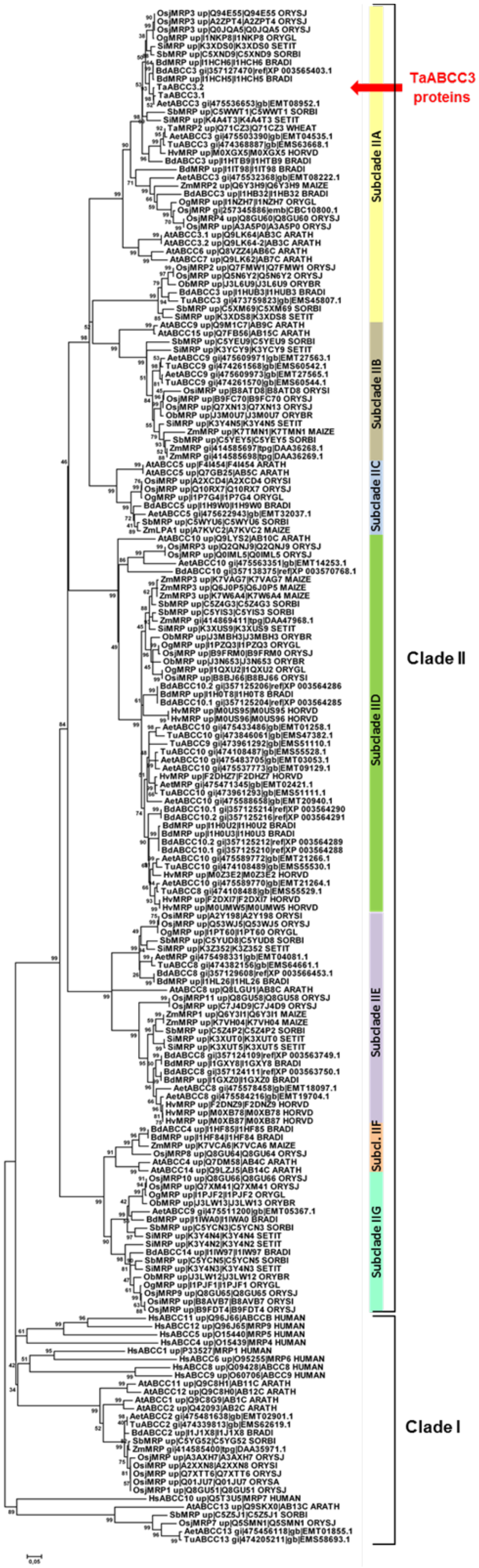
TaABCC3
proteins
Subclade IIA
Subclade IIB
Subclade IIC
Subclade IID
Subclade IIE
Subcl. IIF
Subclade IIG
Clade II
Clade I
0,05

**Supplemental Figure S3: Evolutionary relationships of 187 ABCC-type ABC transporters.** For inferring the evolutionary relationship of the wheat *Ta*ABCC3.1 and *Ta*ABCC3.2 proteins, 185 other full-length ABCC-type ABC transporters, representing proteins from 12 monocot plant species and for reference regarding protein function also proteins from humans (*Homo sapiens*) (N=9) and from the eudicot plant species *Arabidopsis thaliana* (N=17), were subjected to phylogenetic analysis. A dendrogram was constructed in MEGA4 (Tamura *et al.*, 2007), after calculation of Dayhoff evolutionary distance matrix, bootstrap values (1000 replicates) and an un-rooted Neighbor-Joining tree from a multiple protein alignment generated with the Cobalt tool at NCBI (Papadopoulos and Agarwala, 2007). Bootstrap values are shown next to branches and depict the percentage of replicate trees in which associated proteins clustered together in the bootstrap test (1000 replicates). For bootstrap values <50 branches are collapsed. The tree is drawn to scale, i.e. branch lengths correlate directly with the Dayhoff evolutionary distances used to infer the phylogenetic tree. Species name abbreviations: Aet- *Aegilops tauschii* (Tausch's goatgrass); At- *Arabidopsis thaliana* (Mouse-ear cress); Bd- *Brachypodium distachyon* (purple false brome); Hs- *Homo sapiens* (Human); Hv- *Hordeum vulgare* subsp. *vulgare* (barley); Ob- *Oryza brachyantha* (-); Og- *Oryza glaberrima* (African rice); Osi- *Oryza sativa* Indica Group (Indian rice); Osj- *Oryza sativa* Japonica Group (rice); Sb- *Sorghum bicolor* (sorghum); Si- *Setaria italica* (foxtail millet); Ta- *Triticum aestivum* (wheat); Tu- *Triticum urartu* (-); Zm- *Zea mays* (corn). Protein abbreviations: ABCC- ABC transporter C family protein; MRP- Multidrug resistance-associated protein ABC transporter. Protein abbreviations reflect the annotated or assigned protein name as par the current status in the UniProtKB database (The UniProt Consortium, 2012) [or if not available in GenBank (Benson *et al.*, 2013)]. Any proteins previously lacking an abbreviated name were named MRP, after the presence of protein and transmembrane domains required for classification as ABCC-type ABC transporters had been validated. Except for the two newly described *Ta*ABCC3 proteins (highlighted with a red arrow), all protein abbreviations are followed by their respective protein identifiers in the UniProtKB (up) or GenBank (gi) database. Major clades (clade I and II) are indicated and in accordance with published nomenclature. Subclades IIA to IIG (demonstrated with coloured boxes) within clade II were proposed, based on the phylo-genetic clustering and annotation of ABCC proteins within a cluster.

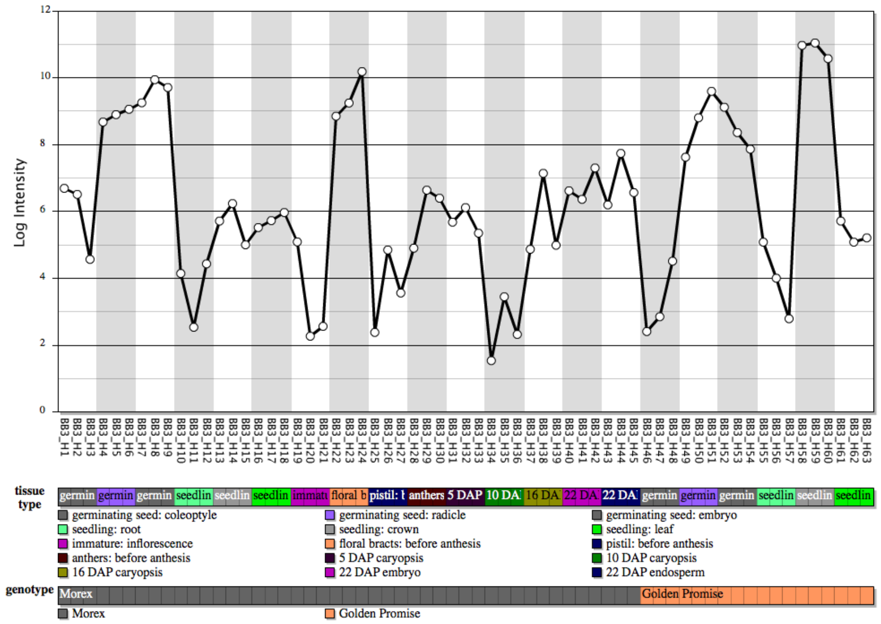


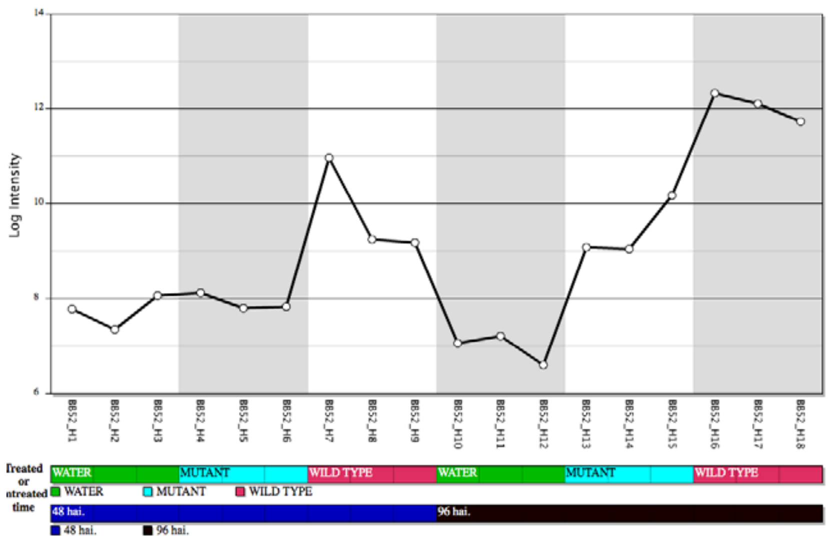


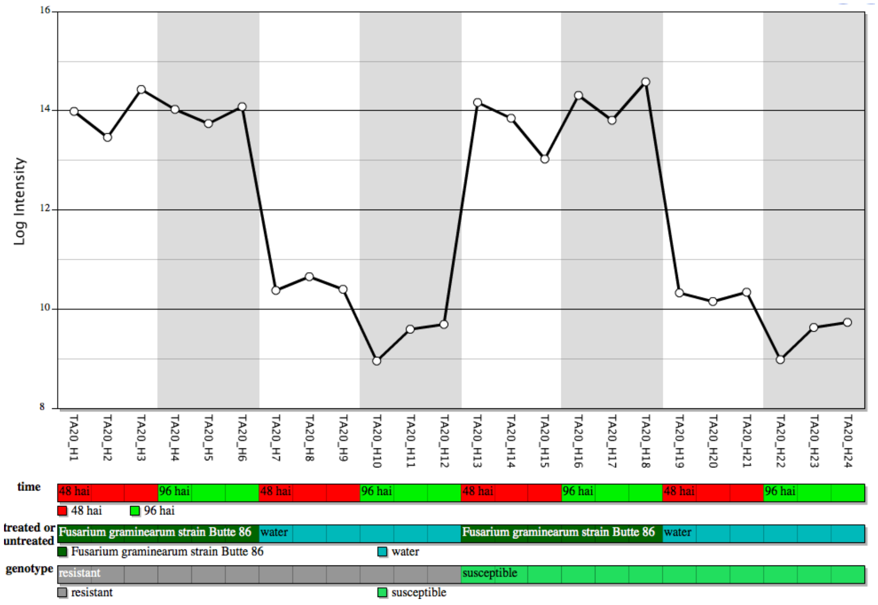


**Supplemental Fig. S4** *In silico* analysis of *TaABCC3* transcript levels (**A**) during development in wheat (**B**) in response to *Fusarium graminearum* in wheat and (**C**) in response to trichothecene production in barley. A and B are respectively based on data for probe Ta.27443.1.S1_at (94.7% identity to *TaABCC3*.1). (A) is taken from the studies conducted by Schreiber *et al*. (2009) using wheat cultivar Chinese Spring. (B) is taken from the study conducted by Jia *et al*. (2009) using near-isogenic line pair carrying resistant and susceptible alleles at the wheat Fhb1 locus. (C) is based on data for probe Barley1_09422 (95.3% identity to *TaABCC3.1*) in the studies conducted by Boddu *et al*. (2007) using a wild type strain of *F. graminearum* and a trichothecene-minus mutant derivative. See relevant papers and the PLEX database for further explanation of the underlying experiments. Graphs were retrieved from the Plant Expression (PLEX) database (www.plexdb.org).

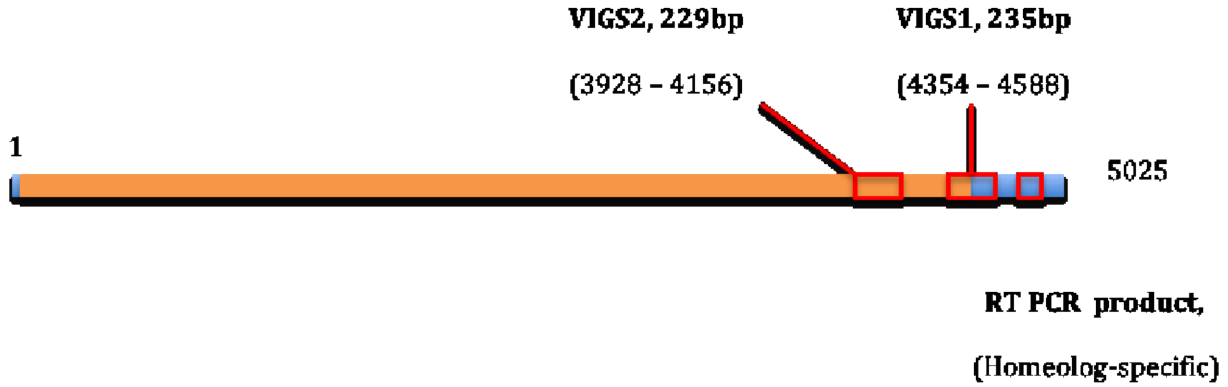


**Supplemental Figure S5: Illustration of the position of the fragments within the mRNA encoding the wheat *TaABCC3.1* ABC transporter on wheat chromosomes 3BS targeted for gene silencing and RT-PCR for virus-induced gene silencing (VIGS) studies.** VIGS fragment 1 (TaABCC3V1) and 2 (TaABCC3V2) did overlap neither each other nor the RT-PCR product. RT-PCR assays were used that were specific to the chromosome 3A, 3B and 3D homeologs. The open reading frame is coloured in orange, the untranslated regions at the 5' and 3' end are coloured in blue. Numbers indicate bp positions in the TaABCC3.1 mRNA sequence (GenBank accession: KM458975).

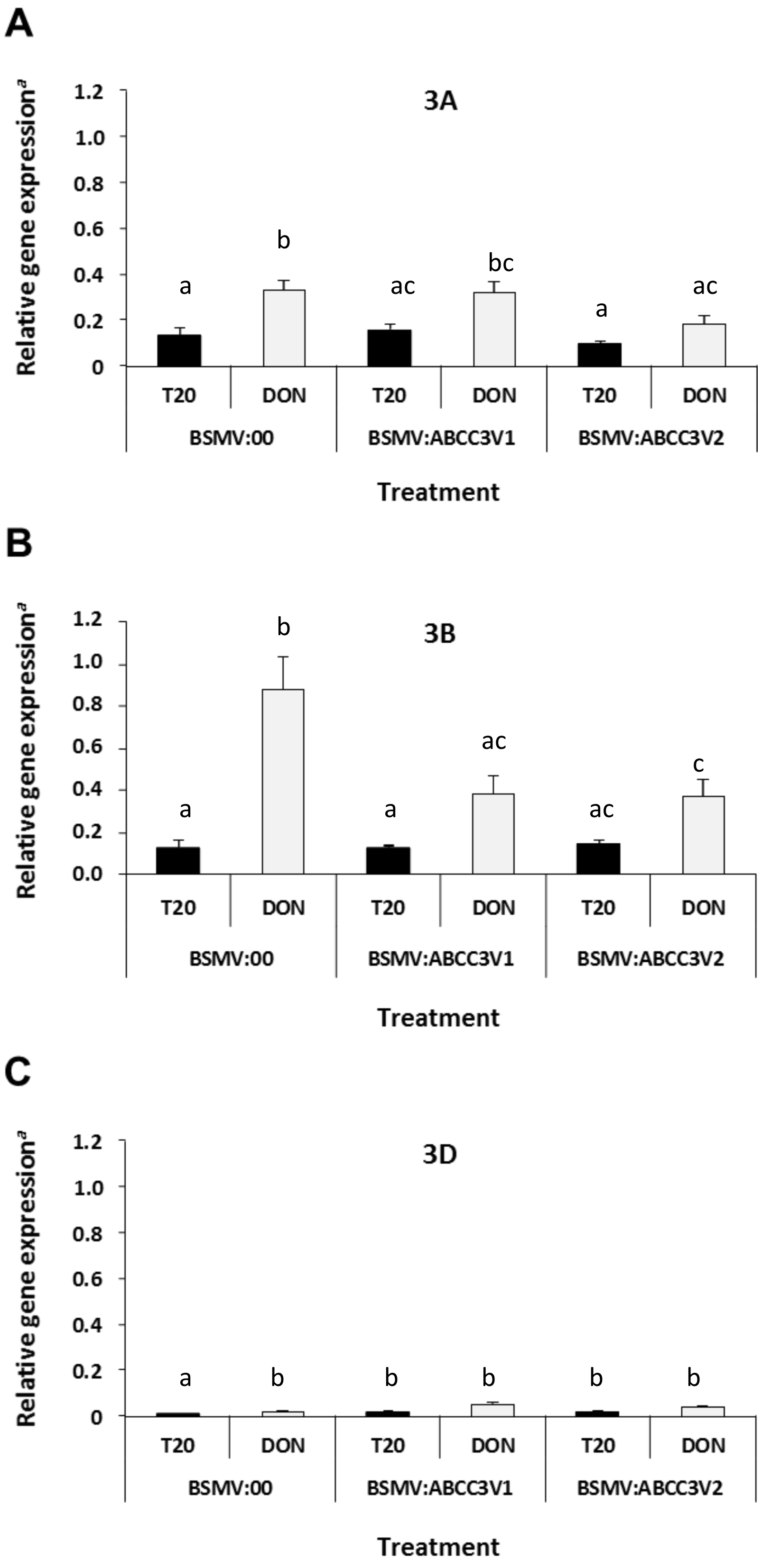

A
3A
Relative gene expression[a]
1.2
1.0
0.8
0.6
0.4
0.2
0
a
b
ac
bc
a
ac
T20
DON
T20
DON
T20
DON
BSMV:00
BSMV:ABCC3V1
BSMV:ABCC3V2
Treatment
B
3B
Relative gene expression[a]
1.2
1.0
0.8
0.6
0.4
0.2
0.0
a
b
a
ac
ac
c
T20
DON
T20
DON
T20
DON
BSMV:00
BSMV:ABCC3V1
BSMV:ABCC3V2
Treatment
C
3D
Relative gene expression[a]
1.2
1.0
0.8
0.6
0.4
0.2
0
a
b
b
b
b
b
T20
DON
T20
DON
T20
DON
BSMV:00
BSMV:ABCC3V1
BSMV:ABCC3V2
Treatment

**Supplemental Figure S6: Analysis of the effect of virus-induced gene silencing (VIGS) of *TaABCC3* in wheat on the transcription of chromosome 3A, 3B and 3D gene variants in mock and deoxynivalenol-treated wheat heads.** Flag leaves of deoxynivalenol (DON) - tolerant wheat cv. CM82036 were rub-inoculated before the emergence of heads (growth stage 47; Zadoks et al., 1974) with *in vitro* transcribed RNAs representing either the empty vector BSMV:00 (empty vector), or one of the two constructs BSMV:ABCC3V1, or BSMV:ABCC3V2 (two constructs containing independent fragments of the chromosome 3B gene homeolog *TaABCC3.1*). At mid-anthesis the central spikelets were treated with either 5 mg $ml^{-1}$ deoxynivalenol (DON) or Tween20 (T20). The specificity of the gene silencing was examined using primers specific to the putative gene homeologs on wheat chromosomes 3A (**A**), 3B (**B**) or 3D (**C**), respectively, and expression of those gene variants was quantified by real time RT-PCR relative to that of the RNA helicase housekeeping gene [$2^{\wedge(-CT\ target)}/2^{\wedge(-CT\ RNA\ helicase)}$]. Variant-specific RT-PCR assays were designed using wheat genomic sequence. Abbreviations: DON-deoxynivalenol, T20- Tween20, BSMV- barley stripe mosaic virus. Bars indicate standard error of the mean calculated from 26-32 heads per treatment combination (at least 10 heads per experiment).

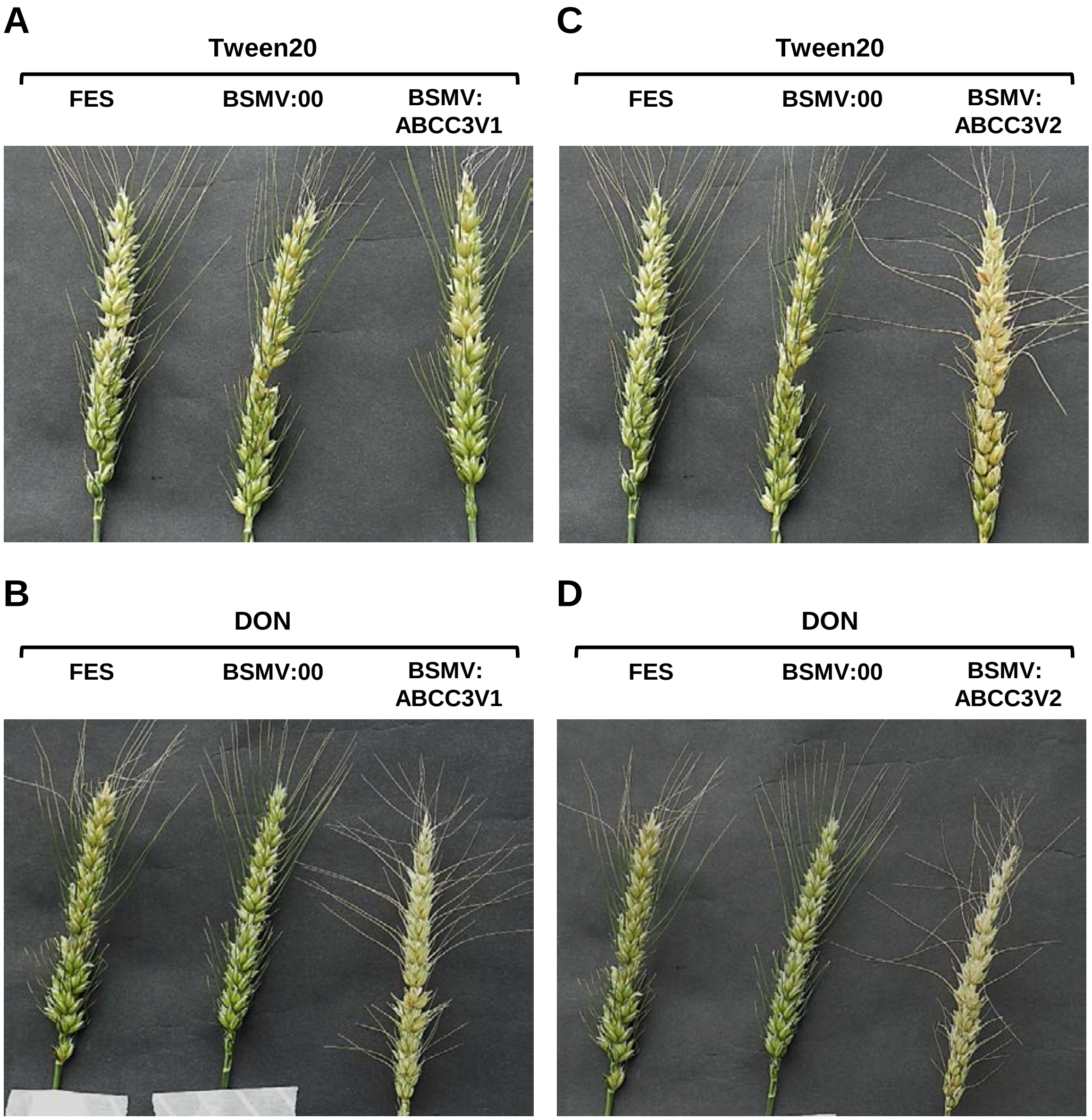


**Supplemental Figure S7: Effect of virus-induced gene silencing (VIGS) of *TaABCC3* genes on ripening in response to either DON or Tween20 treatment.** Flag leaves of deoxynivalenol (DON) -tolerant wheat cv. CM82036 were rub-inoculated before the emergence of heads (growth stage 47; Zadoks *et al.*, 1974) with the VIGS buffer FES, or *in vitro* transcribed RNAs representing either BSMV:00 (empty vector), BSMV:TaABCC3V1, or BSMV:TaABCC3V2 (two constructs containing independent fragments of the *TaABCC3.1* gene). At mid-anthesis the central spikelets were treated with either 5 mg $ml^{-1}$ DON or Tween20. Response of plants treated with either FES, BSMV:00 or BSMV:ABCC3V1 to (**A**) Tween20 or (**B)** DON. Response of plants treated with either FES, BSMV:00 or BSMV:ABCC3V2 to (**C**) Tween20 or (**D)** DON. Photographs were taken at 21 days post-toxin treatment.

## REFERENCES


**Aller SG, Yu J, Ward A, Weng Y, Chittaboina S, Zhuo R, Harrell PM, Trinh YT, Zhang Q, Urbatsch IL, Chang G**. 2009. Structure of P-Glycoprotein Reveals a Molecular Basis for Poly-Specific Drug Binding. *Science* **323**, 1718-1722.

**Benson DA, Cavanaugh M, Clark K, Karsch-Mizrachi I, Lipman DJ, Ostell J, Sayers EW**. 2013. GenBank. *Nucleic Acids Research* **41**, D36-D42.

**Bernsel A, Viklund H, Hennerdal A, Elofsson A**. 2009. TOPCONS: consensus prediction of membrane protein topology. *Nucleic Acids Research* **37**, W465-468.

**Boddu J, Cho S, Muehlbauer GJ**. 2007. Transcriptome Analysis of Trichothecene-Induced Gene Expression in Barley. *Molecular Plant-Microbe Interactions* **20**, 1364-1375.

**Deeley RG, Westlake C, Cole SPC**. 2006. Transmembrane Transport of Endo- and Xenobiotics by Mammalian ATP-Binding Cassette Multidrug Resistance Proteins. *Physiological Reviews* **86**, 849-899.

**Finn RD, Clements J, Eddy SR**. 2011. HMMER web server: interactive sequence similarity searching. *Nucleic Acids Research* **39**, W29-W37.

**Goujon M, McWilliam H, Li W, Valentin F, Squizzato S, Paern J, Lopez R**. 2010. A new bioinformatics analysis tools framework at EMBL-EBI. *Nucleic Acids Research* **38**, W695-W699.

**Gunnaiah R, Kushalappa AC, Duggavathi R, Fox S, Somers DJ**. 2012. Integrated Metabolo-Proteomic Approach to Decipher the Mechanisms by Which Wheat QTL *Fhb1* Contributes to Resistance against *Fusarium graminearum*. *PLoS ONE* **7**, e40695.

**Jia H, Cho S, Muehlbauer GJ**. 2009. Transcriptome Analysis of a Wheat Near-Isogenic Line Pair Carrying Fusarium Head Blight–Resistant and –Susceptible Alleles. *Molecular Plant-Microbe Interactions* **22**, 1366-1378.

**Jin MS, Oldham ML, Zhang Q, Chen J**. 2012. Crystal structure of the multidrug transporter P-glycoprotein from *Caenorhabditis elegans*. *Nature* **490**, 566-569.

**Linton KJ, Higgins CF**. 2007. Structure and function of ABC transporters: the ATP switch provides flexible control. *Pflügers Archiv - European Journal of Physiology* **453**, 555-567.

**Lopez R, Silventoinen V, Robinson S, Kibria A, Gish W**. 2003. WU-Blast2 server at the European Bioinformatics Institute. *Nucleic Acids Research* **31**, 3795-3798.

**Papadopoulos JS, Agarwala R**. 2007. COBALT: constraint-based alignment tool for multiple protein sequences. *Bioinformatics* **23**, 1073-1079.

**Roy A, Kucukural A, Zhang Y**. 2010. I-TASSER: a unified platform for automated protein structure and function prediction. *Nature Protocols* **5**, 725-738.

**Roy A, Yang J, Zhang Y**. 2012. COFACTOR: an accurate comparative algorithm for structure-based protein function annotation. *Nucleic Acids Research* **40**, W471-W477.

**Sánchez-Fernández R, Davies TGE, Coleman JOD, Rea PA**. 2001. The *Arabidopsis thaliana* ABC Protein Superfamily, a Complete Inventory. *The Journal of Biological Chemistry* **276**, 30231-30244.

**Schreiber A, Sutton T, Caldo R, Kalashyan E, Lovell B, Mayo G, Muehlbauer G, Druka A, Waugh R, Wise R, Langridge P, Baumann U**. 2009. Comparative transcriptomics in the Triticeae. *BMC Genomics* **10**, 285.

**Sievers F, Wilm A, Dineen D, Gibson TJ, Karplus K, Li W, Lopez R, McWilliam H, Remmert M, Soding J, Thompson JD, Higgins DG**. 2011. Fast, scalable generation of high-quality protein multiple sequence alignments using Clustal Omega. *Molecular Systems Biology* **7**, 539.

**Soltanloo H, Khorzoghi EG, Ramezanpour S, Arabi MK, Pahlavani MH**. 2010. The Expression Profile of'Chi-1, Glu-2, Glu-3 and PR1. 2'Genes in Scab-resistant and Susceptible Wheat Cultivars during Infection by'Fusarium Graminearum'. *Plant Omics* **3**, 162.

**Tamura K, Dudley J, Nei M, Kumar S**. 2007. MEGA4: Molecular Evolutionary Genetics Analysis (MEGA) software version 4.0. *Molecular Biology and Evolution* **24**, 1596-1599.

**The UniProt Consortium**. 2012. Reorganizing the protein space at the Universal Protein Resource (UniProt). *Nucleic Acids Research* **40**, D71-D75.

**Walter S, Brennan J, Arunachalam C, Ansari K, Hu X, Khan MR, Trognitz F, Trognitz B, Leonard G, Egan D, Doohan FM**. 2008. Components of the gene network associated with genotype-dependent response of wheat to the *Fusarium* mycotoxin deoxynivalenol. *Functional & Integrative Genomics* **8**, 421-427.

**Zadoks JC, Chang TT, Konzak CF**. 1974. A decimal code for the growth stages of cereals. *Weed Research* **14**, 415-421.
**Zhang Y**. 2008. I-TASSER server for protein 3D structure prediction. *BMC Bioinformatics* **9**, 40.
